# Discovery of Phthalazine Derivatives as New PARP1 Inhibitors Through 3D-QSAR, Molecular Docking, and MD Studies

**DOI:** 10.5812/ijpr-169970

**Published:** 2026-06-16

**Authors:** Tahereh Sedghamiz, Somayeh Asgharpour Hasan Kiyadeh, Fatemeh Moosavi, Alireza Poustforoosh

**Affiliations:** 1 Shiraz University of Medical Sciences, Shiraz, Iran; 2 University of Turin, Turin, Italy

## Abstract

**Background:**

Poly(ADP-ribose) polymerase 1 (PARP1) is a well-established therapeutic target in cancer owing to its critical role in DNA damage repair. In this study, we combined structure-based and ligand-based computational approaches to identify and optimize phthalazine derivatives as new PARP1 inhibitors.

**Objectives:**

This study aimed to highlight the potential of computationally guided drug discovery in developing novel phthalazine-based PARP1 inhibitors and to support their candidacy as anticancer agents.

**Methods:**

A 3D-QSAR model was generated using Phase and demonstrated strong statistical performance (R^2^ = 0.89, Q^2^ = 0.701). ADME/T predictions further confirmed the drug-like properties of the optimized compounds. A set of intrinsic PARP1 inhibitors was docked using Glide XP, and detailed interaction analyses were performed. To further validate the docking results, molecular dynamics (MD) simulations were conducted on the top three compounds.

**Results:**

Contour map analysis indicated that steric, electrostatic, hydrogen-bond donor/acceptor, and hydrophobic fields play important roles in determining biological activity. Favorable and unfavorable regions near functional groups such as carbonyls, amines, and aromatic rings provided guidance for designing more potent derivatives. Several newly designed phthalazine derivatives showed improved docking scores and formed more stabilizing interactions than reference inhibitors such as fluzoparib. Among them, compounds 42 and 63 displayed the strongest binding affinities and robust interaction profiles, including multiple conserved hydrogen bonds and π–π stacking interactions. The MD trajectories demonstrated stable binding within the PARP1 active site, with persistent hydrogen bonds, particularly those with Ser904 and Gly863, supporting the reliability of the docking and QSAR findings.

**Conclusions:**

The integrated QSAR, docking, and MD approaches provide strong evidence for the potential of phthalazine derivatives as novel PARP1 inhibitors and identify promising candidates for further experimental validation in anticancer drug discovery.

## 1. Background

Cancer remains a major global health problem. Each year, approximately 20 million people are diagnosed with cancer and nearly 10 million die from the disease, according to the International Agency for Research on Cancer (1). This alarming incidence underscores the urgent need for more effective and selective therapeutic strategies. Although conventional treatment modalities such as surgery, radiotherapy, and conventional chemotherapy have evolved over time, their nonspecific nature often results in substantial toxicity to healthy tissues (2). Recent advances have shifted the standard of care from nonspecific cytotoxic chemotherapy to more targeted treatment modalities that minimize damage to healthy cells and improve patient outcomes (3, 4). Among these modern therapeutic approaches, immunotherapy and targeted therapy have received particular attention. Immunotherapy, especially immune checkpoint blockade targeting PD-1, PD-L1, and CTLA-4, has produced durable clinical responses in several cancer types and has become part of standard or first-line treatment regimens for selected malignancies (5). In parallel, targeted therapies aim to interfere with specific molecular alterations or signaling pathways essential for tumor growth and survival, thereby offering greater selectivity than conventional chemotherapy (6, 7). One important category of targeted anticancer therapy involves disruption of the DNA damage response (DDR), a network essential for maintaining genomic stability and repairing DNA lesions. Within this network, Poly(ADP-ribose) polymerase (PARP) comprises a group of at least 17 enzymes associated with DNA repair pathways (8). PARP-1, the most abundant and well-characterized isoform, has emerged as a promising molecular target in oncology. This 116 kDa nuclear enzyme comprises three functional domains: an N-terminal DNA-binding domain that detects DNA lesions, a central auto-modification domain, and a C-terminal catalytic domain (9). The catalytic domain, also known as the ADP-ribosyltransferase domain, contains a conserved PARP motif responsible for binding nicotinamide adenine dinucleotide (NAD^+^), which serves as the substrate for ADP-ribose transfer reactions (10). This domain facilitates the transfer of ADP-ribose units from NAD^+^ to specific target proteins, including PARP1 itself, a process essential for recruiting and coordinating DNA repair factors (11).

Targeting PARP1 impairs the DNA repair machinery, resulting in persistent DNA damage and, ultimately, cell death. This effect is particularly pronounced in tumors harboring defects in homologous recombination repair (HRR), such as those carrying mutations in the BRCA1 or BRCA2 genes. This therapeutic concept, known as synthetic lethality, exploits the dual impairment of DNA repair pathways to selectively eliminate cancer cells. On this basis, four PARP inhibitors, olaparib, rucaparib, niraparib, and talazoparib, have gained clinical approval for the treatment of BRCA-mutated, HER2-negative advanced, metastatic ovarian, or breast cancer (12).

Although current PARP1 inhibitors represent a significant advance in targeted cancer therapy, challenges such as acquired resistance, off-target toxicities, and limited effectiveness in HR-proficient tumors remain substantial barriers to long-term therapeutic success (13, 14). These challenges highlight the ongoing need to discover and optimize next-generation PARP1 inhibitors with improved selectivity, efficacy, and safety profiles. Among the diverse chemical scaffolds explored, phthalazine derivatives have garnered considerable attention owing to their favorable pharmacokinetic characteristics and structural compatibility with the PARP1 catalytic domain. Preclinical evaluations have demonstrated that several phthalazine-based compounds exhibit strong inhibitory activity against PARP1, underscoring the importance of further elucidating their structure–activity relationships (SAR) to guide rational design and optimization (15, 16). Other compounds reported as PARP1 inhibitors include imidazobenzodiazepines (17), quinazolinones and quinoxaline derivatives (18), substituted uracil derivatives (19), adenosine substituted isoindolones (20), phenantridin-6-ones (21), and naphtyridin-6-ones (21, 22). Imidazobenzodiazepines were introduced as potent PARP1 inhibitors with ionizable groups designed to improve pharmaceutical properties. Quinazolinone and quinoxaline derivatives are important bioisosteric scaffolds; quinazolinones have shown relatively higher PARP1 selectivity, whereas quinoxalines may favor PARP2 inhibition. More recent quinazolinone analogues achieved low-nanomolar PARP1 inhibition, with compound 12c showing an IC_50_ of approximately 30.38 nM, comparable to olaparib (23). Substituted fused uracil derivatives improved from micromolar activity to IC_50_ values below 20 nM after structural optimization, highlighting the value of hydrogen-bond-rich heterocycles in the PARP1 NAD^+^ pocket (19). Adenosine-substituted isoindolones also showed nanomolar potency when appropriate linkers were used, with representative compounds displaying IC_50_ values of 45 and 100 nM (20). Phenanthridin-6-one derivatives provided an early planar tricyclic PARP inhibitor scaffold, but poor aqueous solubility limited their development, whereas naphthyridin-6-one analogues were designed to improve potency, solubility, and pharmacokinetic properties (24). Collectively, these scaffolds demonstrate the importance of conserved hydrogen-bonding, aromatic stacking, and NAD^+^-mimetic interactions in PARP1 inhibition, while also emphasizing the need for continued optimization of potency, selectivity, and drug-like properties.

Computational methods play a critical role in accelerating drug discovery by enabling the rational design and screening of drug candidates in silico. Structure-based approaches, such as molecular docking and molecular dynamics, predict interactions between ligands and target proteins using 3D structural data (25-29). Ligand-based methods, including QSAR and pharmacophore modeling, rely on known active compounds to identify new candidates (30). Virtual screening and ADMET predictions further streamline hit identification and optimize pharmacokinetic profiles (31). The integration of machine learning has enhanced these approaches, improving prediction accuracy and enabling large-scale compound evaluation (32).

Quantitative structure–activity relationship (QSAR) modeling is a robust computational strategy for establishing correlations between molecular descriptors and the biological activity of compounds. This approach facilitates the prediction of activity for novel chemical entities, thereby supporting structure-based drug design and optimization (33). In the context of PARP1 inhibition, QSAR models enable the identification of key structural features that influence binding affinity and activity, thereby expediting the drug discovery pipeline by minimizing reliance on resource-intensive experimental screening (34).

Ramadan et al. (2020) synthesized a series of quinazolinone-based compounds as PARP1 inhibitors and performed docking, QSAR, and in silico ADMET modeling. Compound 12c showed promising activity (IC_50_ ≈ 30 nM, similar to olaparib) and cell-cycle arrest in MCF-7 cells (23). Another study reported the design and synthesis of phthalazinone derivatives, evaluating their PARP1 inhibitory activity via in vitro assays. The authors also conducted molecular docking and SAR analyses, identifying key substituent effects on potency (16). One study described in vitro PARP1 inhibition (and intracellular PARylation assays) of a phthalazinone-based series. Several compounds demonstrated strong activity against BRCA2-deficient Capan-1 cells (16). A significant study modeled 51 PARP1 inhibitors using GA-MLR and LS-SVM, achieving excellent internal validation metrics (Q^2^_cv_ = 0.971, R^2^ = 0.977) and integrating docking-derived descriptors to enhance predictive power (35). A 2024 modeling effort developed MLR and SVM models using four molecular descriptors on phthalazinone derivatives. The models achieved high predictivity (MLR r^2^ = 0.944, Q^2^_cv_ = 0.921; SVM r^2^ = 0.947, Q^2^_cv_ = 0.887). Docking revealed interactions with key residues (GLY227, MET229, PHE230, TYR246) that mimicked the binding modes of known PARP inhibitors such as olaparib. Top candidates were validated via 200-ns MD simulations, confirming binding stability (36). The recent PARP1 activity study (2024) applied SMOTE, KNN, SHAP, and matched molecular pair analysis specifically to the phthalazinone scaffold. This interpretable ML-QSAR framework improved prediction quality and identified transformation rules to guide medicinal chemistry (37). Another study has combined docking, ADMET profiling, virtual screening, MD, and QSAR for robust PARP1 inhibitor discovery (36).

Phthalazine/phthalazinone scaffolds are structurally analogous to the known PARP1 inhibitor olaparib, forming key interactions in the active site (e.g. with Gly863/Ser904) (36).

## 2. Objectives

In this study, we developed and validated QSAR models to investigate the inhibitory activity of a series of phthalazine derivatives against PARP1. Using statistical and docking approaches, we aimed to identify key molecular descriptors that govern bioactivity and to propose optimized structures with enhanced potency. The insights gained from this work are expected to support the rational design of novel PARP1 inhibitors with improved therapeutic potential in cancer treatment.

## 3. Methods

### 3.1. Dataset Collection and Preparation

A dataset of 30 phthalazine derivatives, along with their experimental IC_50_ values, was retrieved from the PubChem database (21). The selected compounds are phthalazine derivatives with documented experimental IC_50_ values. The dataset was chosen to encompass a diverse range of chemical modifications within the phthalazine scaffold. The final selection was constrained by the availability of compounds meeting these criteria while keeping the dataset manageable for QSAR modeling. The SMILES, 2D structures, and activities of the compounds are shown in Table S1 in Supplementary File. The IC_50_ values were converted to pIC_50_ (-log_10_ IC_50_) to standardize activity representation. The dataset was randomly split into two parts: 80% for model training and 20% for testing, for model development and external validation, respectively.

Ligands were processed using the LigPrep module of the Schrödinger Suite (Schrödinger Release 2025 - 3: LigPrep, Schrödinger, LLC, New York, NY, 2025). During ligand preparation, the original chirality was retained, neutral states were generated at physiological pH (7.0 ± 0.5), and a single low-energy conformer was generated for each molecule using the OPLS4 force field. The aligned ligands are shown in Figure S1 in Supplementary File.

### 3.2. Protein Preparation and Grid Generation

The crystal structure of PARP1 in complex with the co-crystallized ligand veliparib (PDB ID: 7KK6) was downloaded from the Protein Data Bank (PDB). The Protein Preparation Wizard in Maestro was used to prepare the protein (38), including assigning bond orders, adding hydrogens, optimizing H-bond networks, and minimizing the structure under the OPLS4 force field.

The Receptor Grid Generation panel in Maestro (Schrödinger) was used to generate the receptor grid. The co-crystallized ligand was used to define the active site, and the grid box was centered accordingly. The grid box was centered on the bound ligand and typically spans approximately 20 × 20 × 20 Å for the outer box and 10 × 10 × 10 Å for the inner box, with grid center dimensions of X = 15.0, Y = 32.5, Z = 18.7 used for flexible ligand sampling. The grid center corresponds to the centroid of the reference ligand. All docking runs were performed using these default settings under the XP algorithm, which confines ligand sampling to the defined binding pocket. Default Van der Waals scaling values were applied, with a scaling factor of 0.8 and a partial charge cutoff of 0.15.

### 3.3. 3D-QSAR Model Generation (Field-Based QSAR)

A field-based 3D-QSAR model was constructed using the Phase module in Schrödinger (39). The aligned dataset of phthalazine derivatives (Figure S1 in Supplementary File) was used to compute field descriptors, including steric, electrostatic, and hydrophobic contributions.

Partial Least Squares (PLS) regression was used to establish a quantitative relationship between the computed molecular fields and experimental pIC_50_ values. The optimal number of PLS components was determined via leave-one-out (LOO) cross-validation within the training set. The model was subsequently validated using the test set and assessed using standard statistical parameters such as R^2^, Q^2^, RMSE, and the Pearson correlation coefficient. The Schrödinger Suite (Schrödinger, LLC, New York, NY, USA) used in this work was accessed through a licensed academic installation available at our collaborating research facility. All calculations were performed in accordance with the software’s academic licensing terms.

### 3.4. Activity Prediction and Virtual Screening

The 13 intrinsic inhibitors of PARP1 were docked into the prepared receptor using Glide (extra precision mode) (40) to confirm key interactions and active site accommodation (Table 1). A focused library of 6560 phthalazine derivatives was assembled by querying the PubChem database for compounds containing the phthalazine scaffold and its derivatives. The compounds were downloaded in SDF format, filtered to remove duplicates and structurally inconsistent entries, and subsequently prepared using Schrödinger LigPrep to ensure consistency in protonation state and stereochemistry. The 6560 phthalazine derivatives were predicted for activity using the developed QSAR model, and the top predicted actives with higher activities than the most active compound in our dataset were selected for molecular docking studies. All docking simulations were carried out in Glide-XP mode using the same grid parameters described earlier. The protein–ligand complexes were evaluated based on activities, docking scores, and interaction profiles.

**Table 1.** The Statistical Data of Predicted Model

Variables	Values
**SD**	0.276
**R2**	0.890
**RMSE**	0.160
**Q2**	0.701
**Pearson-R**	0.977
**MAE**	0.128
**Adjusted R2**	0.870
**F Value**	71

### 3.5. ADME/T Analysis

Following the molecular docking analysis, ADME/T (Absorption, Distribution, Metabolism, Excretion, and Toxicity) profiling was performed for the most active compounds to assess their pharmacokinetic properties. This evaluation provides insight into the drug-likeness and potential of these molecules for subsequent in vitro and in vivo studies. The ADME/T analysis was performed using the QikProp module in the Schrödinger suite (41).

### 3.6. Molecular Dynamics Simulations

Compounds with high predicted activity, favorable docking scores, and multiple key interactions with critical residues in the PARP1 active site were selected for molecular dynamics simulations. Independent and unbiased molecular dynamics (MD) simulations were performed for each compound in complex with the protein to confirm the stability of the selected compounds and to rationalize their relative potencies. This approach enabled detailed monitoring of the compounds’ influence on receptor conformation, as well as the specific intermolecular interactions formed. Each system was solvated in a cubic box of TIP3P (42) water molecules and neutralized by adding Na^+^ and Cl⁻ ions to reach a physiological salt concentration of 150 mM. All simulations were carried out using GROMACS 2023.3 (43) with a 2 fs integration time step. Pressure was maintained semi-isotropically at 1 bar via the C-rescale barostat (44) with a coupling constant of 5 ps, while temperature was regulated at 300 K using the V-rescale thermostat (45) with a coupling constant of 0.1 ps. Van der Waals interactions were modeled using the Lennard-Jones potential with a cutoff radius of 1.2 nm and a smooth switching function applied between 1.0 and 1.2 nm, without dispersion correction. Electrostatics were treated using the Particle Mesh Ewald (PME) method with a real-space cutoff of 1.2 nm. The protein and ions were described by the AMBER99SB-ILDN force field (46), while ligands were parameterized with the Biki Life Sciences software package (47). Partial atomic charges were assigned via the RESP method based on density functional theory (DFT) calculations, and topologies were generated using ACPYPE (48), which provided parameters for bonds, angles, and dihedrals. Hydrogen bond constraints were applied using the LINCS algorithm (49). Prior to production runs, each system underwent two steps of steepest-descent energy minimization using the configurations of the compounds obtained from docking until a maximum force below 10 kJ/mol was achieved (50). This was followed by six consecutive 100 ps NVT equilibration steps with gradually increasing temperature up to 300 K and a subsequent 1 ns NPT equilibration. Periodic boundary conditions were used for all MD simulations. The equilibrated systems were then simulated for 100 ns during production runs. The simulations were performed on a Linux-based high-performance workstation using 16 parallel CPU cores, which is a standard configuration for medium-scale MD simulations.

## 4. Results

### 4.1. Field-Based 3D-QSAR Model, Validation, and Visualization

A field-based 3D-QSAR model was successfully developed using Gaussian field descriptors and partial least squares (PLS) regression with four components. The model incorporated steric, electrostatic, hydrogen bond donor (HBD), hydrogen bond acceptor (HBA), and hydrophobic fields to elucidate the structural features influencing biological activity. Model visualization was performed using contour maps, which highlight favorable and unfavorable regions for each field type. The model demonstrated strong statistical performance, with a coefficient of determination (R^2^) of 0.89 and a predictive correlation coefficient (Q^2^) of 0.701, indicating high reliability and predictive capability. Additional validation parameters, including root mean square error (RMSE), standard deviation (SD), and Pearson’s correlation coefficient, further supported the robustness of the model. A summary of the statistical parameters is provided in Table 2. The contour maps illustrate key features of how the compound interacts with the protein, highlighting molecular regions important for shape and biological activity. These maps aid in identifying which parts of the molecule should be modified to design new compounds with stronger or weaker interactions, depending on whether the regions are favorable or unfavorable. The steric contour analysis of the most active compound (Figure 1A) reveals key spatial features influencing biological activity. In the active molecule (Figure 1A), the green contour on the succinimide group favors bulky substituents; adding larger groups in this region is predicted to increase biological activity.

**Table 2.** The Intrinsic Inhibitors of PARP1 Along with Their Docking Scores and Interactions

Compound Name	Docking Score	Residue	Interaction
**AZD9574**	-6.285	SER904, GLY863; TYR907	Hbond; π-π
**Fluzoparib**	-10.827	SER904, GLY863, GLN759; TYR907	Hbond; π-π
**Iniparib**	-5.820	SER904, GLY863T; YR907	Hbond; π-π
**Niraparib**	-4.683	TYR907	π-π
**NMS-03305293**	-7.561	SER904, GLY863	Hbond
**Olaparib**	-9.699	SER904, GLY863; TYR907	Hbond; π-π
**Pamiparib**	-8.571	SER904, GLY863; TYR907	Hbond; π-π
**Rucaparib**	-7.108	SER904, GLY863, GLU763; TYR907	Hbond; π-π
**SC10914**	-4.649	SER724; TYR907	Hbond; π-π
**Saruparib**	-5.581	GLY888, GLU763; TYR896	Hbond; π-π
**Stenoparib**	-9.482	SER904, GLY863; TYR907	Hbond; π-π
**Talazoparib**	-9.986	SER904, GLY863; TYR907	Hbond; π-π
**Veliparib**	-7.957	SER904, GLY863, GLU763; TYR907	Hbond; π-π

**Figure 1.**
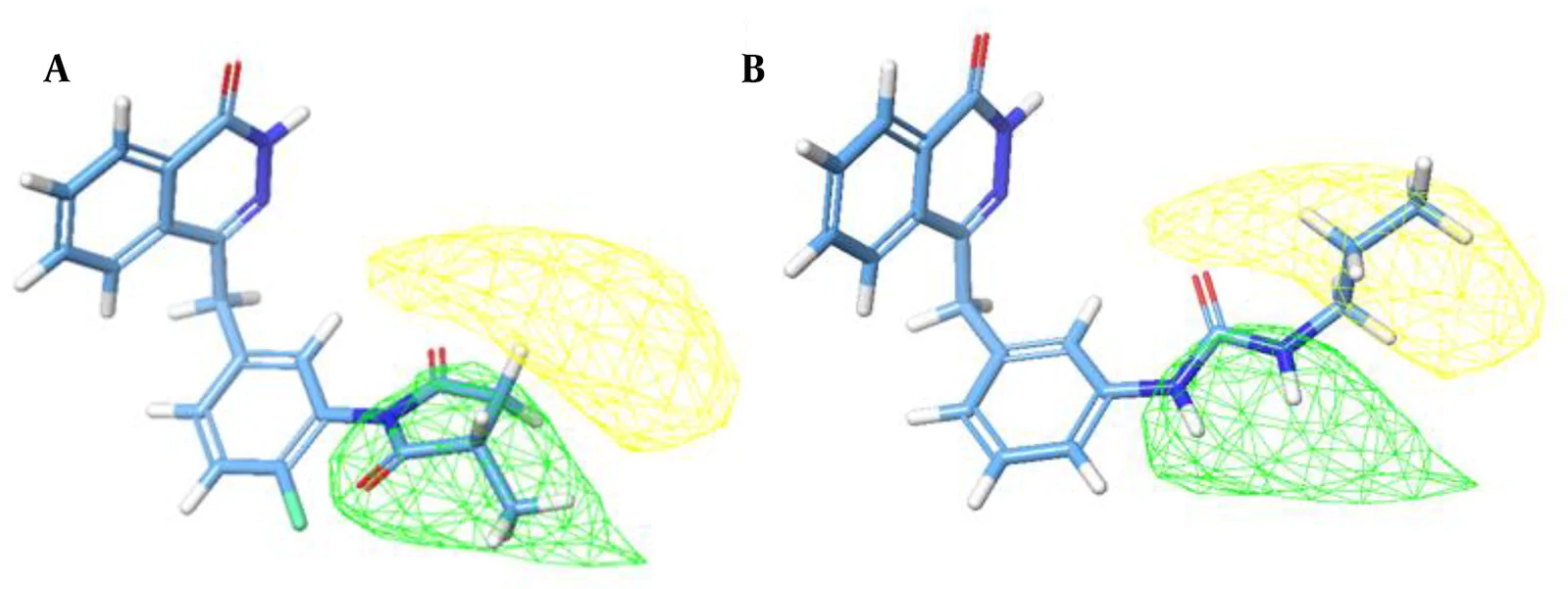
Contour maps from the 3D-QSAR model illustrating steric fields for active(A) and inactive (B) compounds PARP1 inhibitors. Green contours indicate regions where bulky groups enhance activity; yellow contours indicate regions where bulk is unfavorable

Figure 1 Contour maps from the 3D-QSAR model illustrating steric fields for active(a) and inactive (b) compounds PARP1 inhibitors. Green contours indicate regions where bulky groups enhance activity; yellow contours indicate regions where bulk is unfavorable

Increasing steric bulk (larger alkyl or aryl groups) around this area would likely improve potency. The yellow contour is sterically disfavored; therefore, adding bulk in this region may cause steric clashes or reduce binding affinity. Thus, the yellow contour above the upper part of the molecule indicates that bulky groups in this region would likely decrease biological activity. In the inactive molecule (Figure 1B), the yellow contour, which indicates a less favorable region, is located on the alkyl chain, suggesting that alkyl groups in this region reduce activity. The hydrophobic contour map (Figure 2A) shows a yellow contour near the top of the succinmimide ring close to the CH2 group and around the central ring, suggesting that adding hydrophobic bulk, such as methyl, ethyl, or aromatic groups, at these positions could enhance binding affinity or potency.

**Figure 2.**
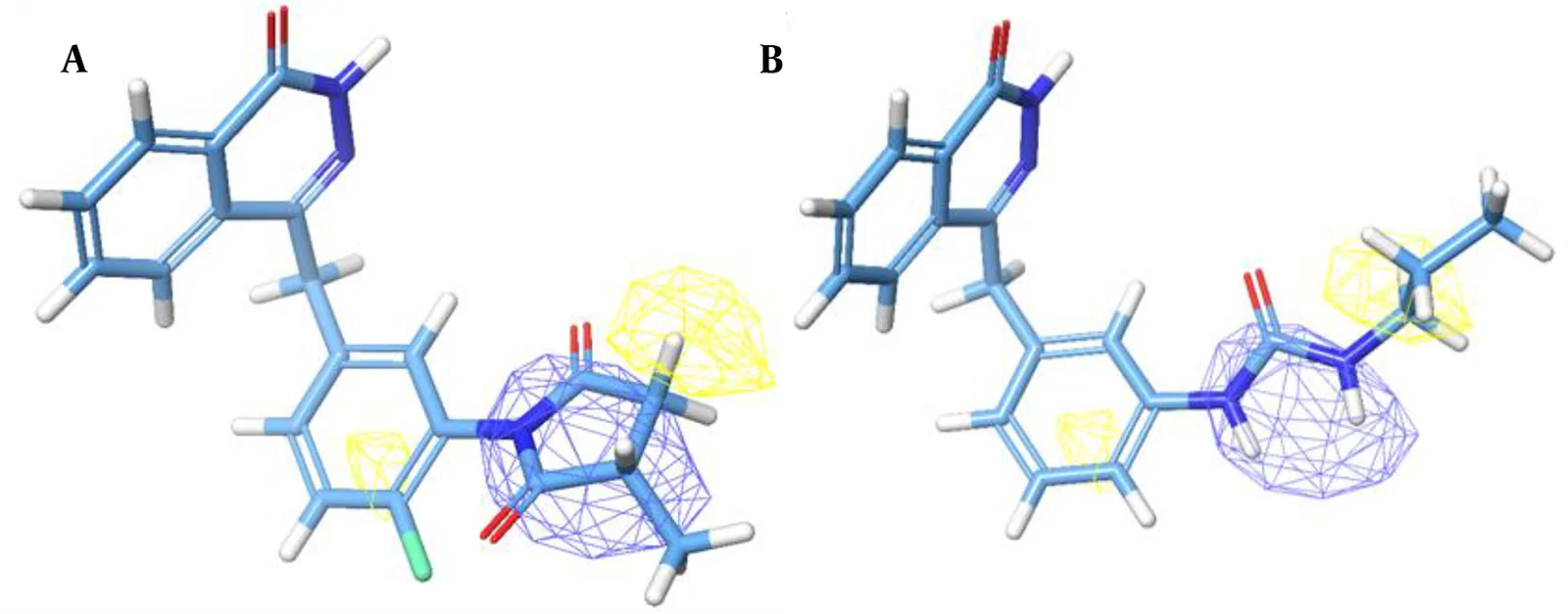
Contour maps from the 3D-QSAR model illustrating electrostatic fields for active (A) and inactive (B) compounds PARP1 inhibitors. Blue contours show areas where electropositive groups are favorable; red contours show regions where electronegative groups improve activity

The blue contour around the succinimide ring and near the carbonyl region suggests that introducing hydrophilic groups such as OH, NH2, or carbonyl, or reducing nonpolar bulk, would improve activity. Figure 2B shows the hydrophobic contour map for the inactive molecule. The yellow contour near the central ring and terminal side chain indicates that adding nonpolar or bulky hydrophobic groups, such as alkyl, aryl, and halogen substituents, in these regions would likely enhance activity. The blue contour surrounding the carbonyl region shows that increased hydrophilic character in these positions enhances activity. This suggests that the hydrophobic contour map shows similar effects for both active and inactive molecules. The electrostatic contour map of the active molecule (Figure 3A) shows blue contours near the succinimide group, indicating that a positive electrostatic potential on the ligand increases activity, whereas the red contour near the carbonyl and chlorine atom represents regions where a negative electrostatic potential on the ligand increases activity. A positively charged or electropositive patch on the ligand at the blue contour positions can form favorable electrostatic contacts with electron-rich or negatively charged features of the binding site. In the red contour region, the model predicts increased activity when the ligand presents negative electrostatic potential in this volume. For the inactive molecule (Figure 3B), the blue contours represent regions where electropositive substituents enhance activity, mainly located near the amine and carbonyl groups of the side chain. This indicates that introducing or strengthening positive electrostatic potential in these regions would be favorable for increasing activity. In contrast, a red contour, signifying a region where electronegative substituents are preferred, is observed near the central aromatic ring. This implies that the presence of electron-withdrawing or electronegative groups, such as halogens, carbonyl, or nitro substituents, in this region would enhance binding affinity.

**Figure 3.**
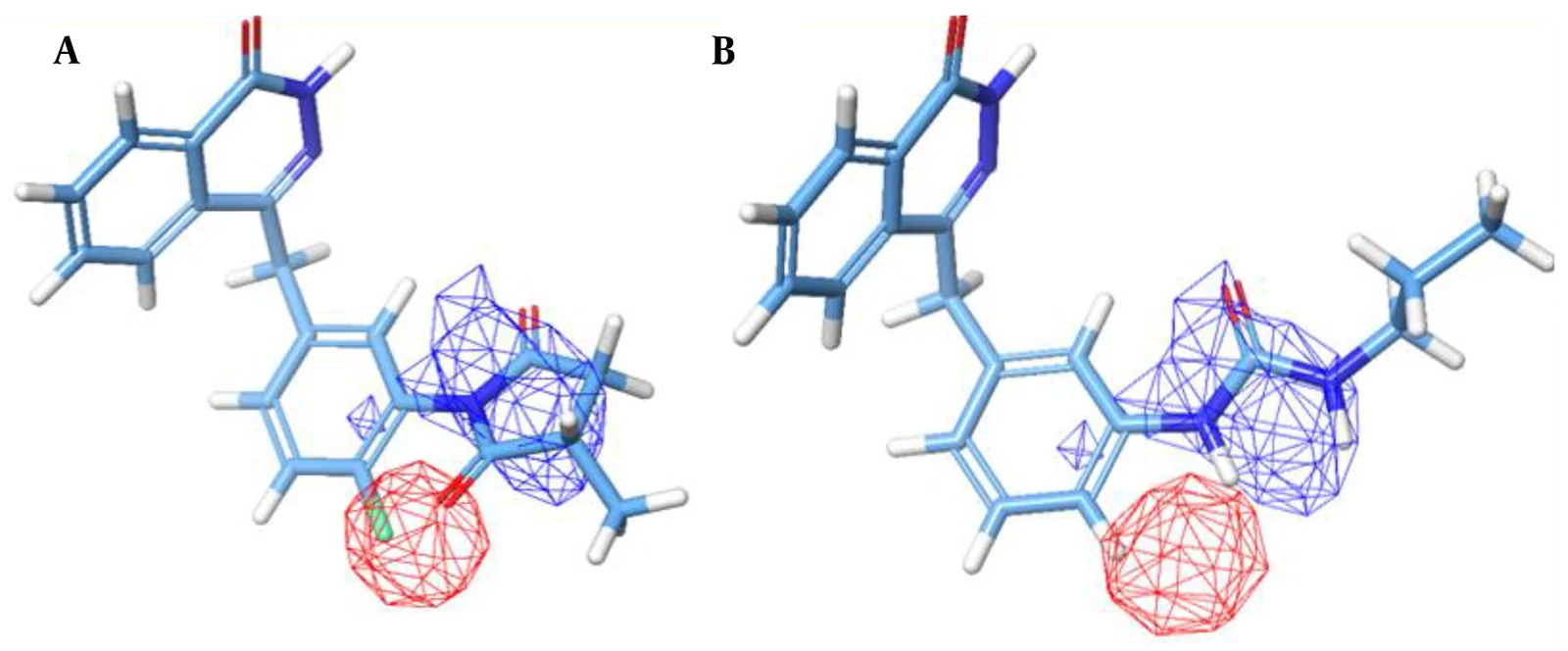
Contour maps from the 3D-QSAR model illustrating hydrogen bond acceptor (HBA) fields for active (A) and inactive (B) compounds PARP1 inhibitors. Red contours show favorable acceptor regions; magenta contours indicate positions where acceptors reduce activity

The hydrogen bond acceptor contour map of the active molecule (Figure 4A) reveals spatial regions that favor or disfavor hydrogen bond acceptor features for enhancing biological activity. The red contours indicate regions where strong hydrogen bond acceptors contribute positively to activity, whereas magenta contours denote regions where such features negatively influence binding affinity. A prominent red contour is observed near the carbonyl group and the chlorine atom, suggesting that the presence of electron-rich acceptor sites in this region facilitates favorable interactions with complementary donor residues in the receptor environment. Conversely, the magenta contour is located near the upper region of the succinimide moiety, indicating that introducing or maintaining hydrogen bond acceptor functionality in this region would be unfavorable for biological activity.

**Figure 4.**
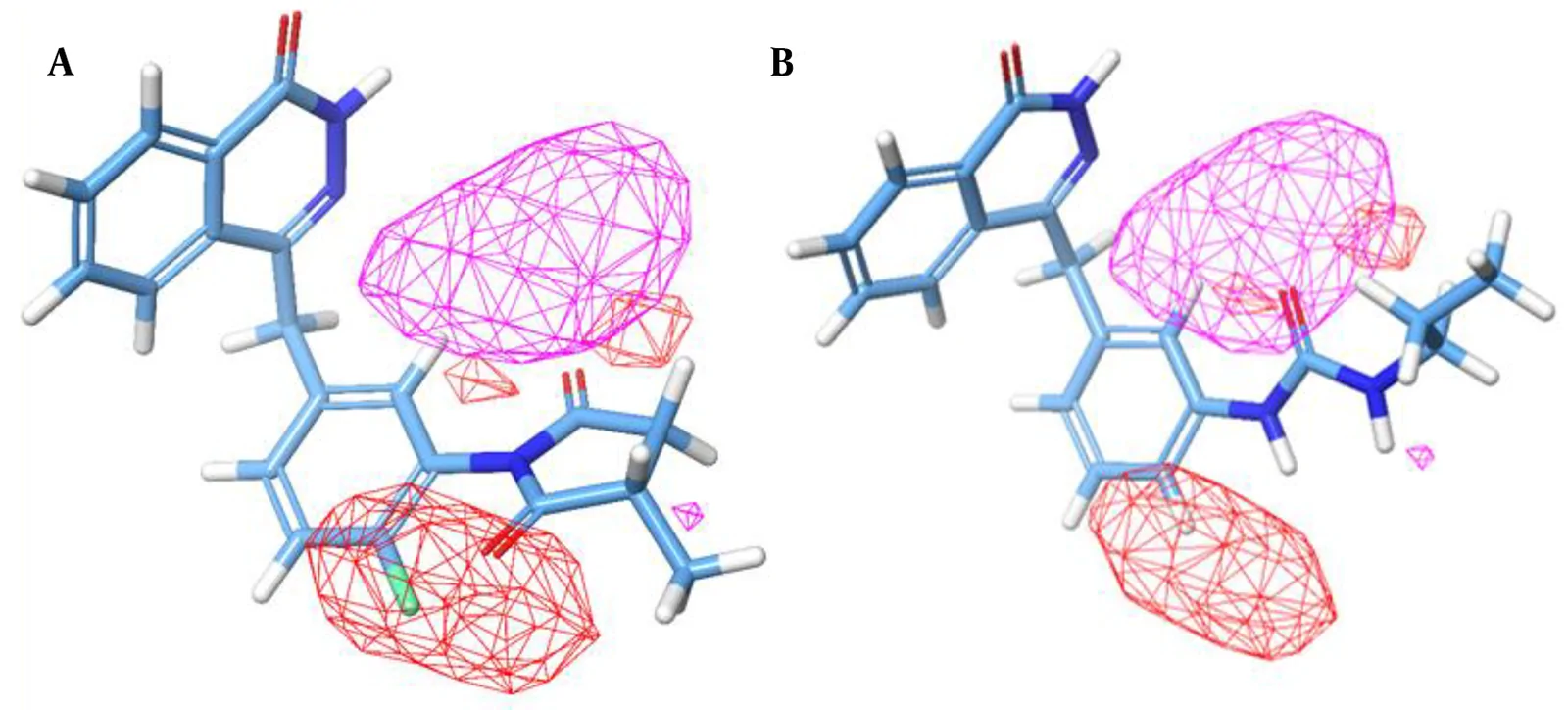
Contour maps from the 3D-QSAR model illustrating hydrogen bond donor (HBD), fields for active (A) and inactive (B) PARP1 inhibitors. Blue-violet contours represent favorable positions for hydrogen bond donors; cyan contours indicate unfavorable donor regions.

The hydrogen bond acceptor contour map of the inactive molecule (Figure 4B) shows both favorable and unfavorable regions for hydrogen bond acceptor features. The red contours represent regions where the presence of a hydrogen bond acceptor group (such as oxygen or nitrogen atoms with lone pairs) would have a positive effect on biological activity. In this molecule, red contours are observed near the benzene ring and a smaller one close to the alkyl chain. These regions suggest that adding or strengthening hydrogen bond acceptor groups at these positions could enhance the molecule’s interaction with the receptor, possibly improving activity.

In contrast, the magenta contour represents a negative effect for hydrogen bond acceptors, indicating that placing such groups in this region would reduce activity. This magenta contour is located near the carbonyl group, suggesting that an acceptor in this area may disrupt favorable interactions or cause unfavorable electrostatic effects in the receptor binding site.

Overall, the distribution of these contours indicates that, relative to the active compound, the inactive molecule has hydrogen bond acceptor groups positioned in less favorable regions, which could explain its lower biological activity.

The hydrogen bond donor contour map for the active molecule (Figure 5A) illustrates regions where donor groups contribute positively or negatively to biological activity. The blue-violet contour, located near the amine group of the fused aromatic ring, represents a region where hydrogen bond donor groups are favorable. This indicates that the presence of a hydrogen bond donor (such as an –NH or –OH group) in this position can enhance binding interactions with the receptor, most likely by forming stabilizing hydrogen bonds with acceptor residues in the binding site. In contrast, the cyan contour, positioned below the succinimide group near the carbonyl region, indicates an unfavorable region for donor groups. Introducing or maintaining hydrogen bond donors in this region could weaken receptor binding, possibly due to steric clashes or an inappropriate electronic environment that does not support hydrogen bond formation.

**Figure 5.**
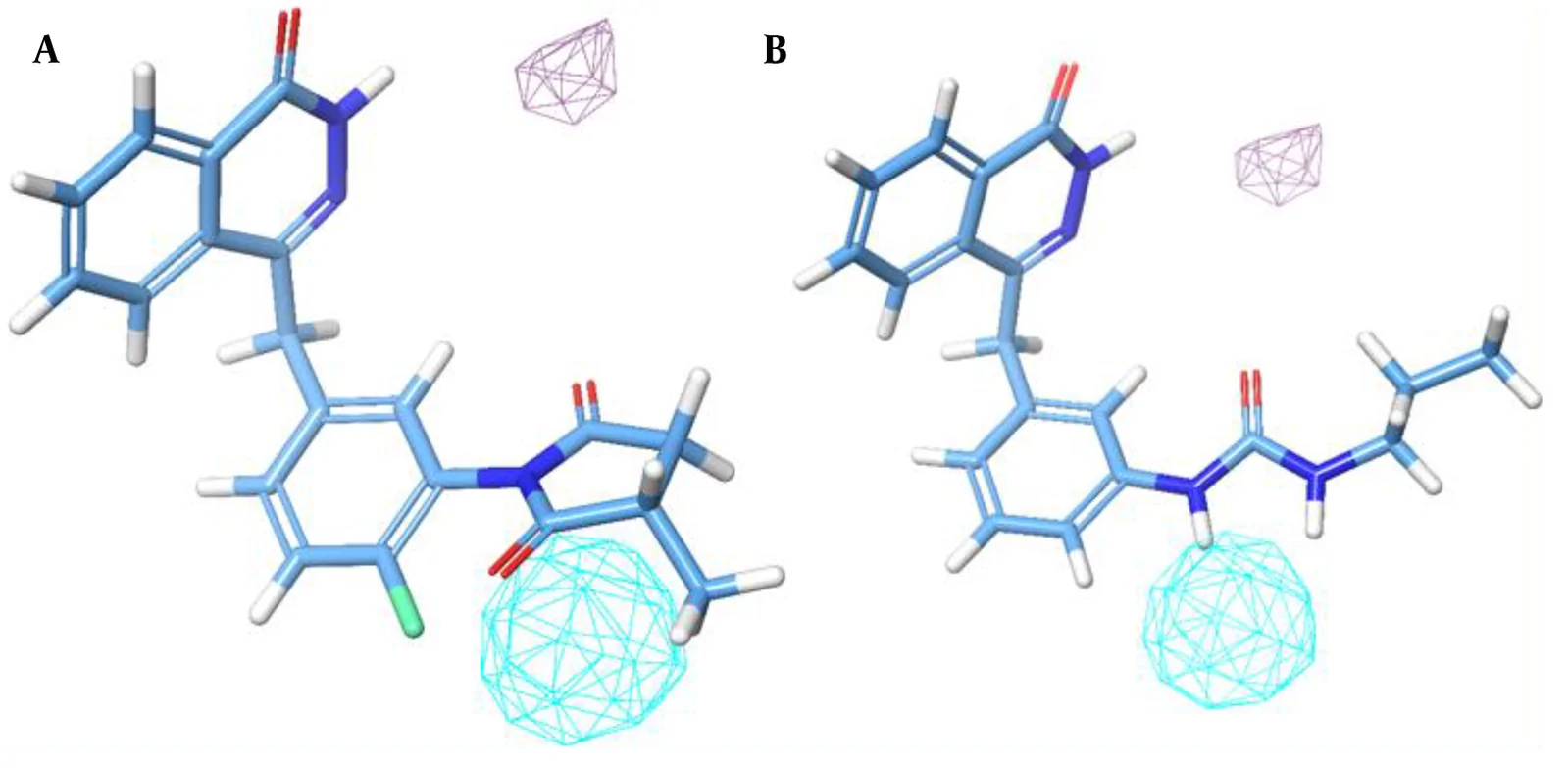
The presence of compound 42 among different residues of PARP1. Yellow dashed line indicates the hydrogen bond and blue dashed lines are Pi interactions in 3D illustration

The hydrogen bond donor contour map for the inactive molecule (Figure 5B) shows how donor group placement affects biological activity. The blue-violet contour, positioned near the amine group of the fused aromatic ring, marks a region where hydrogen bond donor groups are favorable, suggesting that adding or maintaining donor groups in this region could improve interactions with the receptor. The cyan contour, located near the amine groups, represents regions where donor groups are unfavorable. The presence of donors in these regions could reduce activity, possibly due to steric hindrance or repulsive interactions within the receptor binding pocket.

A total of 5,650 phthalazine derivatives were virtually screened using our QSAR model. Among them, 181 compounds exhibited predicted activities greater than the highest observed activity in the training dataset (pIC_50_ = 8.39). The top predicted activities among these 181 compounds were 9.269, 9.094, and 9.004. The most active candidates were docked into the PARP1 binding site. Docking scores and predicted activities for the top 20 active compounds are summarized in Table 3, and their 2D structures are depicted in Table S2 in Supplementary File.

**Table 3.** The Top 20 Active Compound Obtained from Docking Along with Their Docking Score, Activities and Interactions

Name	Docking Score	Activity	Residue	Interaction
**Compound 1**	-9.554	9.269	SER904; GLY863; MET890; TYR907	Hbond; Hbond; Hbond; π-π
**Compound2**	-5.995	9.094	GLU988; HIE909; TYR896; TYR907	Hbond; Hbond; π-π; π-π
**Compound3**	-5.238	9.004	HIE909; TYR896	Hbond; π-π
**Compound5**	-9.269	8.981	GLU763; TYR907; TYR896	Hbond,salt bridge; π-π, π-cation; π-cation
**Compound6**	-8.242	8.894	MET890; GLY863; SER904; TYR896; TYR907	Hbond; Hbond; Hbond; π-π; π-π
**Compound7**	-9.069	8.884	SER904; GLY863; TYR907	Hbond; Hbond; π-π
**Compound8**	-6.425	8.816	SER904; MET890; GLN759	Hbond; Hbond; Hbond
**Compound9**	-10.701	8.748	SER904; GLY863; TYR907; MET890; TYR907	Hbond; Hbond; Hbond; Hbond; π-π
**Compound10**	-4.446	8.743	MET890	Hbond
**Compound12**	-5.291	8.729	MET890	Hbond
**Compound13**	-11.387	8.725	GLY863; SER904; MET890; TYR907	Hbond; Hbond; Hbond; Hbond, π-π
**Compound14**	-5.176	8.711	GLU988; HIE909; TYR907	Hbond; Hbond; Salt-bridge
**Compound15**	-8.440	8.687	GLY863; SER904; MET890; TYR907	Hbond; Hbond; Hbond; π-π
**Compound16**	-4.250	8.679	TYR907; TYR896	Hbond; π-π
**Compound17**	-10.097	8.668	GLY863; SER904; MET890; TYR907	Hbond; Hbond; Hbond; π-π
**Compound18**	-2.514	8.659	GLY863; SER904; MET890; TYR907	Hbond; Hbond; Hbond; π-π
**Compound19**	-6.366	8.635	HIE909	Hbond
**Compound20**	-5.503	8.612	GLN759; TYR907	Hbond; π-π
**Compound42**	-12.145	8.448	GLY863; SER904; TYR907; ASN906; GLN759; GLU763; TYR907; TYR896; HIP862	Hbond; Hbond; Hbond; Hbond; Hbond; Hbond; π-π; π-π; π-Cation
**Compound63**	-11.469	8.336	GLY863; SER904; TYR907; GLN759; TYR907	Hbond; Hbond; Hbond; Hbond; π-π

### 4.2. Docking Study

The most active compound from the training set was subjected to molecular docking within the PARP1 active site and yielded a docking score of −10.139. This compound formed key hydrogen bonds with GLY863 and SER904, along with an additional hydrogen bond with TYR907, and established three π–π stacking interactions involving TYR907 and TYR896.

The reliability of the docking procedure was assessed by evaluating the similarity between the predicted lowest-energy binding pose, determined by the Glide XP scoring function, and the experimentally observed conformation from X-ray crystallography. In this study, validation was performed by redocking the co-crystallized ligand veliparib into the active site of the PARP1 protein (PDB ID: 7KK6). The resulting docked pose showed excellent alignment with the ligand’s crystallographic orientation. A root mean square deviation (RMSD) of 2.0 Å between the docked and experimental conformations confirmed the capability of Glide XP to accurately reproduce the binding geometry, thereby supporting its reliability for structure-based studies of PARP1 inhibitors.

The docked compounds were numbered according to activity from 1 to 181. Analysis of the docking poses revealed that the most active ligands consistently formed hydrogen bonds with key residues GLY863 and SER904, which are also involved in interactions with the co-crystallized reference ligand (Table 3). Additional interactions, such as hydrogen bonds with MET890 and π–π stacking with TYR907, were observed in several cases. For example, Compound 6 formed the two critical hydrogen bonds with GLY863 and SER904 via its carbonyl and amine groups, in addition to an interaction with MET890 and π–π stacking with both TYR907 and TYR896. Compound 7 exhibited a docking score of −9.07 and a predicted activity of 8.88, with hydrogen bonds to GLY863 and SER904 and one π–π interaction with TYR907. Compound 8 (activity: 8.816, docking score: −6.43) formed hydrogen bonds with SER904, MET890, and GLN759 (Table 3).

Compound 9 demonstrated a strong docking score of −10.701 and an activity of 8.748. It established four hydrogen bonds, including the key interactions with GLY863 and SER904, as well as additional hydrogen bonds with TYR907 and MET890, and a π–π interaction with TYR907 (Figure S2 in Supplementary File). Similarly, Compound 13 (docking score: = −11.387, activity: = 8.725) formed the two key hydrogen bonds and additional interactions with TYR907 and MET890, along with two π–π stacking interactions with TYR907 (Figure S3 in Supplementary File). Compound 15, with a docking score of −8.44 and an activity of 8.687, displayed hydrogen bonding to SER904, GLY863, and MET890, and a π–π interaction with TYR907. Compound 17 (docking score: −10.097, activity: 8.668) and Compound 18 (docking score: −2.514, activity: 8.659) both retained the key interactions with GLY863 and SER904, with additional bonding to MET890 and π–π stacking with TYR907 (Table 3).

Figure S2 in Supplementary File shows Compound 9 among different residues of PARP1 and the 2D structures. The yellow dashed line indicates hydrogen bonds, and the blue dashed lines indicate π interactions in the 3D illustration. Among all compounds, Compound 42 exhibited the most favorable docking score (−12.145) and a predicted activity of 8.44. It formed five hydrogen bonds, including the conserved interactions with GLY863 and SER904, and three π–π interactions involving TYR907 and TYR896 (Figure 6). Compound 63 also showed a strong docking score of −11.469 and an activity of 8.336, forming multiple hydrogen bonds beyond the key interactions and two π–π stacking interactions with TYR907 (Figure 7). These results collectively highlight the significance of conserved hydrogen bond interactions with GLY863 and SER904 and π–π interactions with TYR907 in enhancing binding affinity and biological activity.

**Figure 6.**
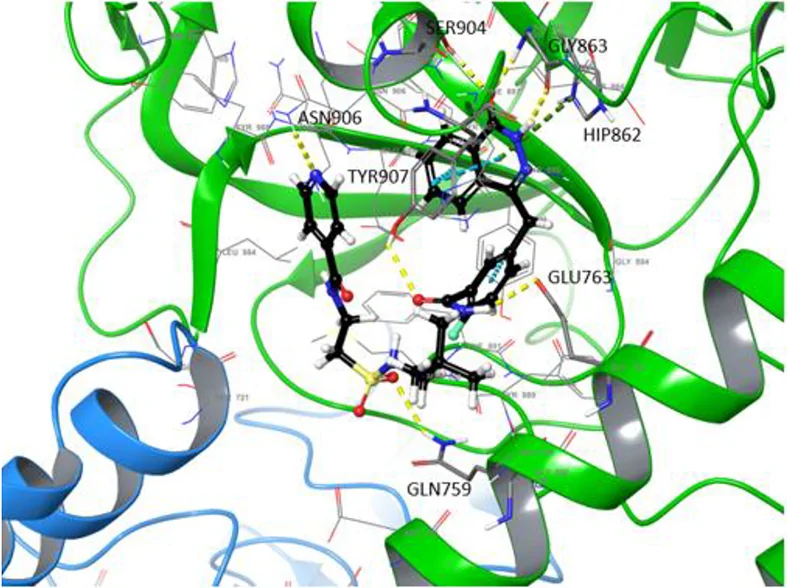
The presence of compound 63 among different residues of PARP1 and the 2D structures.

**Figure 7.**
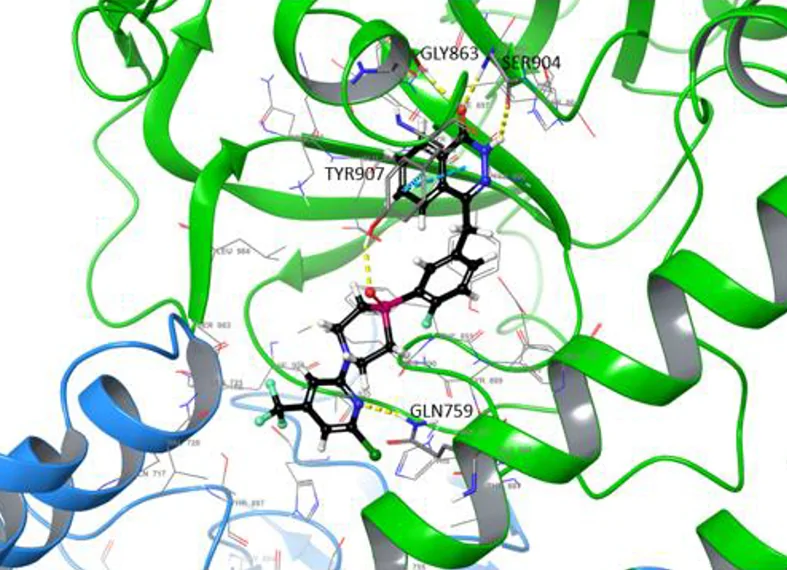
RMSF values of the heavy atoms of the compounds over the 100-ns MD simulations. This quantifies the atomic-level flexibility of each ligand throughout the simulation, highlighting regions of structural rigidity and mobility. CMP1 and CMP63 exhibit relatively low RMSF values, indicating stable and well-anchored binding conformations, whereas CMP42 shows higher fluctuations in specific domains, reflecting greater flexibility consistent with its partially extended binding orientation

The yellow dashed line indicates hydrogen bonds, and the blue dashed lines indicate π interactions in the 3D illustration.

Molecular docking studies were performed on 13 known intrinsic PARP1 inhibitors. The binding interactions are illustrated in Table 2. Among them, fluzoparib exhibited the most favorable docking score (–10.827 kcal/mol), forming key hydrogen bonds with SER904 and GLY863, along with an additional hydrogen bond with GLN759 and a π–π stacking interaction with TYR907. In comparison, the newly identified compounds from our QSAR-guided screening, particularly compounds 42 and 63, demonstrated more favorable docking scores, enhanced activity, and a greater number of stabilizing interactions within the PARP1 binding pocket, indicating enhanced binding affinity relative to the intrinsic inhibitors.

### 4.3. ADME/T Analysis

The ADME/T analysis was conducted for the active compounds listed in Table 3, with the results of four selected compounds summarized in Table 4. Several pharmacokinetic parameters were calculated, including solute molecular weight, which ranged between 130 and 720 g/mol. All compounds exhibited molecular weights within the acceptable range, indicating favorable drug-likeness with respect to this criterion. In addition to molecular weight, hydrogen bond donor and acceptor counts are key descriptors reflecting a compound’s capacity to engage in crucial molecular interactions with protein targets. Two other critical pharmacokinetic parameters include QPPCaco and QPPMDCK, which are predictive of intestinal absorption (gut-blood barrier) and blood-brain barrier permeability, respectively. Optimal values for these parameters typically fall between 25 and 500. Compound 15 exhibited a QPPCaco value below 25, suggesting limited potential for intestinal absorption. Furthermore, the QPPMDCK values for compounds 15 and 63 fell outside the acceptable range, indicating inadequate blood-brain barrier penetration. Lastly, the "Rule of Five" and "Rule of Three" metrics, which assess drug-likeness, indicate improved drug potential when their values are close to zero.

**Table 4.** ADME/T Evaluation of the Top-Performing Compounds

Variables	Compound 15	Compound17	Compound42	Compound63	Reference range
**Solute molecular weight**	463.554	434.494	594.659	550.879	130 - 725
**Solute dipole moment (D)**	4.10	4.16	5.916	2.811	1.0 - 12.5
**SASA**	791.989	742.295	905.774	791.789	300 - 1000
**FOSA**	353.763	336.292	280.819	160.713	0 - 750
**FISA**	169.056	185.404	234.425	144.902	7 - 330
**PISA**	239.782	220.599	377.481	274.341	0 - 450
**WPSA**	0	0	13.489	211.933	0 - 175
**Solute molecular volume(Å3)**	1440.438	1436.502	1712.547	1467.310	500 - 2000
**Solute as hydrogen bond donor**	3.0	1.0	3.0	1.0	0 - 6
**Solute as hydrogen bond acceptor**	8.0	8.5	12.0	8.5	2.0 - 20.0
**Solute globularity(sphere=1)**	0.779	0.794	0.764	0.789	0.75 - 0.95
**QP polarizability(Å3)**	51.214	47.271	58.278	53.904	13.0 - 70.0
**QPlog p for hexadecane/gas**	15.115	13.882	19.244	14.741	4.0 - 18.0
**QPlog p for octanol/gas**	26.169	22.122	31.151	25.361	8.0 - 35.0
**QPlog p for water/gas**	15.808	15.669	19.630	13.947	4.0 - 45.0
**QPlog p for octanol/water**	2.842	2.498	3.612	5.031	-2.0 - 6.5
**QPlog S aqueous solubility**	-4.256	-4.966	-7.394	-8.356	-6.5 - 5.0
**QPlog HERG**	-7.707	-4.536	-7.145	-6.261	<-5.0
**QPPCaco (nm/s)**	15.365	94.288	59.272	418.596	25 - 500
**QPlog BB**	-0.667	-1.636	-2.802	-0.590	-3.0 - 1.2
**QPPMDCK (nm/s)**	9.615	74.201	27.659	2795.911	25 - 500
**QPlog Kp**	-7.532	-3.775	-3.36	-3.032	Kp in cm/h
**IP (eV)**	9.066	9.36	9.414	8.929	7.9 - 10.55
**EA (eV)**	0.891	0.865	0.943	0.877	-0.9 - 1.7
**#metab**	1	3	3	1	1 - 8
**QPlog Khsa**	0.694	0.074	0.311	0.8	-1.5 - 1.5
**Human oral absorption**	2	3	1	1	
**Percent human oral absorption**	64.821	76.909	53.911	77.414	25 - 80
**PSA**	111.244	119.874	182.798	91.879	7 - 200
**Rule of five**	0	0	2	2	Max is 4
**Rule of three**	1	0	1	1	Max is 3

### 4.4. Molecular Dynamics Simulation

Compounds with high predicted activity and favorable docking scores (Compounds 1, 42, and 63) were evaluated by MD simulation. To investigate interaction details, we performed root mean square deviation (RMSD) and root mean square fluctuation (RMSF) analyses on each trajectory to assess the stability of the selected compounds in the binding site. For simplicity, we refer to each simulation as CMP1, CMP42, and CMP63.

Global conformational stability: The RMSD plots in Figure S4 in Supplementary File illustrate structural deviations of the receptor over the course of the simulation, reflecting its overall motion throughout the trajectory. No deviations greater than 0.3 nm were observed, indicating that the compounds did not affect the binding site or overall protein conformation.

Structural stability of the compounds: RMSD values for each compound over the trajectory are shown in Figure S5 in Supplementary File, indicating a higher deviation for CMP42. This did not indicate unbinding over the trajectory; rather, it mainly reflects motion of the compound domain located outside the active site. Based on the initial docking results and H-bond analysis, Ser904 forms highly stable bonds with the compounds and maintains a stable pose in the receptor, as verified by visual inspection and confirmed by long MD simulations. This interaction involves the phthalazine moiety and represents the key interaction. The Root-Mean-Square Fluctuation (RMSF) plots (Figure 8) quantify the flexibility of individual residues in contact with each compound. Protein residues in complexes with CMP1 and CMP63 displayed limited fluctuations in the range of 0.0 - 0.2 nm, whereas slightly higher fluctuations (0.0 - 0.4 nm) were observed for CMP42, consistent with the flexible orientation of its side-chain region. Importantly, residues Gly863 and Ser904, located near the hydroxyl and amine groups of the phthalazine moiety, exhibited low fluctuations, indicating stable hydrogen-bond interactions with the ligands throughout the simulation. Hydrogen-bond interactions were quantified over the entire simulation using the BRIDGE software (51). A total of 10,000 frames sampled at 10 ps intervals were analyzed, excluding water-mediated interactions and focusing only on direct hydrogen bonds. The analysis employed standard geometric criteria: a donor–acceptor distance ≤ 3.5 Å and a donor–hydrogen–acceptor angle ≥ 150°. Occupancy values, calculated as the fraction of simulation frames in which a specific hydrogen bond was maintained, are reported in Figure 9. Across all three complexes, Ser904 and Gly863 were identified as key hydrogen-bonding residues, exhibiting the highest occupancy values. These persistent interactions corroborate the docking observations and emphasize their crucial role in stabilizing ligand binding. The strong and consistent hydrogen bonds formed by CMP1 and CMP63 explain their superior stability, whereas the moderate fluctuations in CMP42 suggest partial flexibility of peripheral groups without loss of binding integrity.

**Figure 8.**
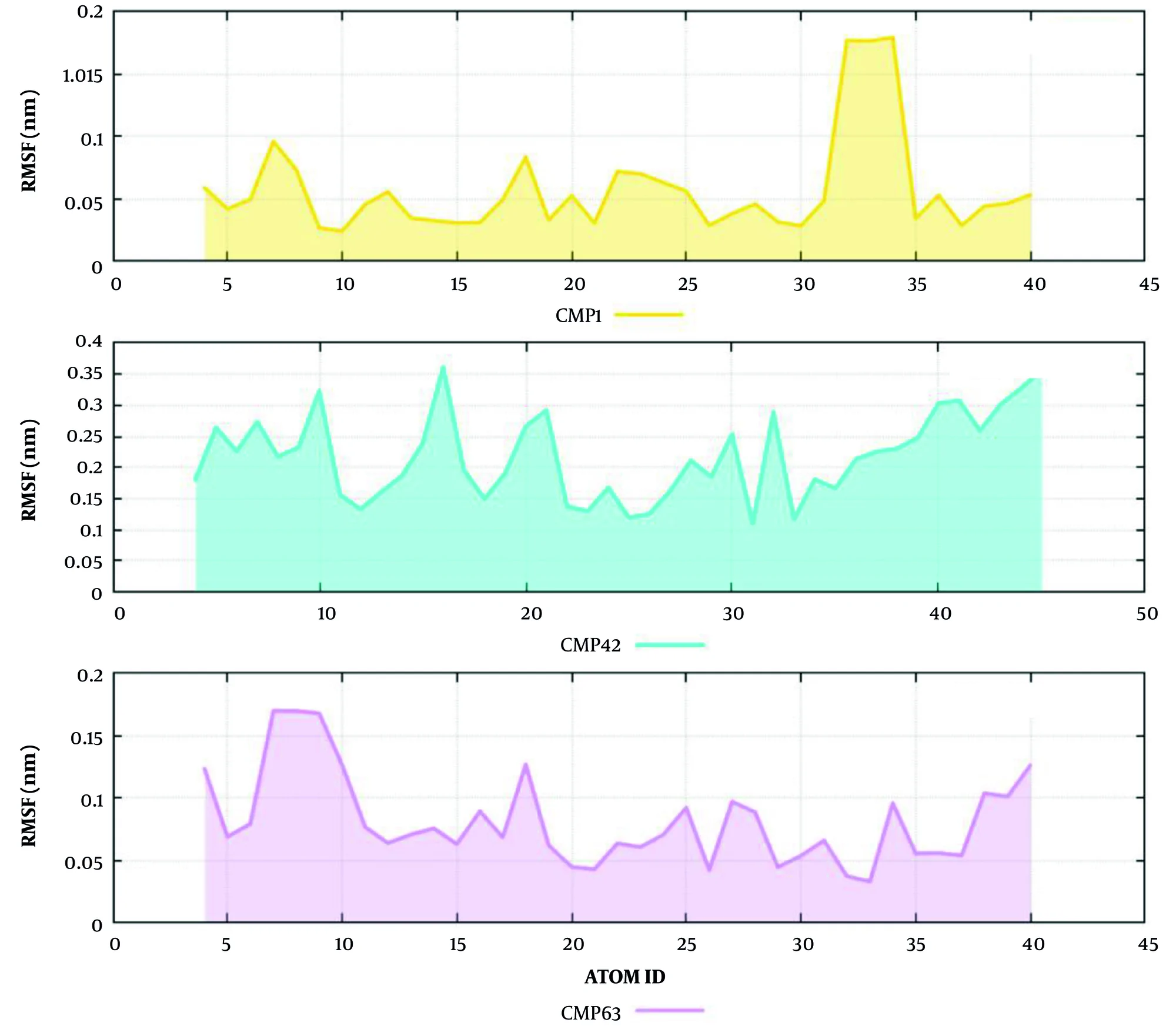
The Hydrogen bond occupancy of the three compounds through the 100-ns MD simulation. The plot displays the percentage of simulation time during which key hydrogen bonds between each ligand and receptor residues are maintained. High occupancy values indicate stable interactions including Ser904 emerging as a major anchoring residue across all compounds. Additional interactions are specific to ligands, such as Arg865 for CMP42 and Gln759 for CMP63, highlight differences in binding modes and contribute to the overall stability of the complexes

**Figure 9.**
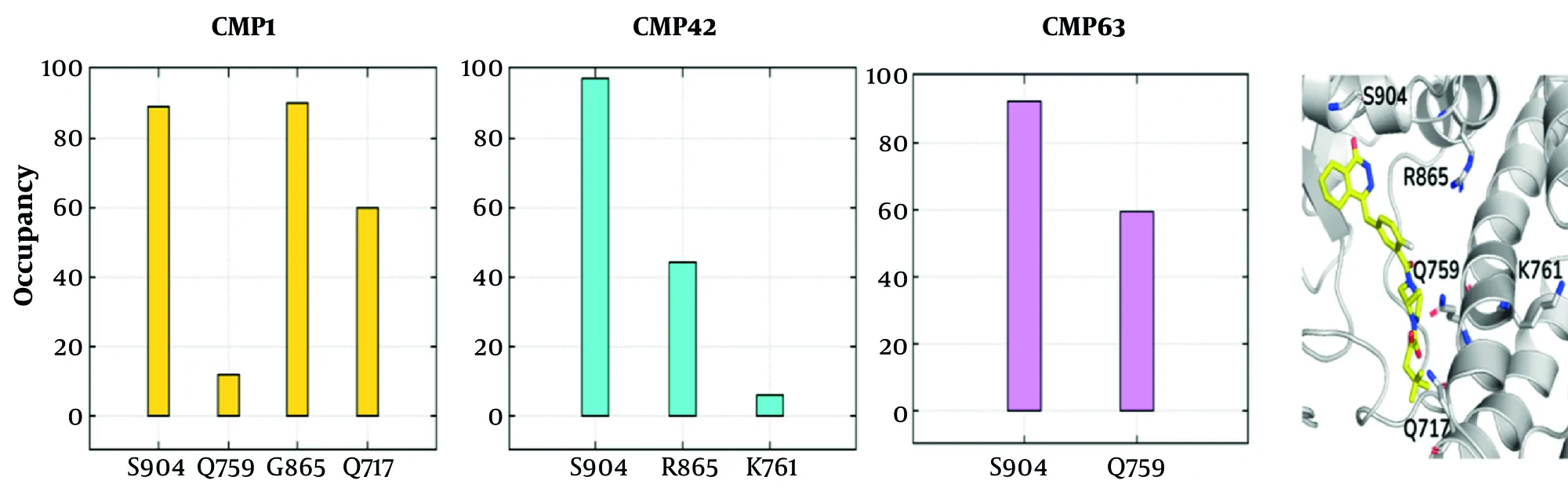
Graphical workflow for identifying phthalazine derivatives as PARP1 inhibitors

## 5. Discussion

Phthalazinone-based scaffolds have emerged as promising pharmacophores for PARP1 inhibition, inspired in part by the structural motif in approved drugs such as olaparib. Structural studies show that the phthalazinone core inserts into the groove-shaped binding pocket of PARP1, forming critical hydrogen bonds with Gly863 and Ser904, while aromatic substitutions such as a 4-fluorobenzyl moiety enhance binding through interactions with Tyr907 and adjacent residues (16). Inhibiting PARP1 is particularly valuable in cancer therapy, as it can induce synthetic lethality in tumor cells with defective homologous recombination repair pathways, such as BRCA-mutated cancers. Despite the clinical success of several PARP1 inhibitors, limitations such as drug resistance, off-target effects, and suboptimal pharmacokinetics underscore the urgent need to develop novel and more selective PARP1-targeted therapies (52). Therefore, continued optimization of phthalazine derivatives may provide a promising route toward more effective and safer anticancer agents. In this context, further optimization of substituents on the phthalazinone core, such as tailored aromatic tails, polar functional groups to improve solubility, or dual-targeting appendages such as dithio or hydroxamic acid motifs, can yield derivatives that show strong PARylation inhibition, potent anti-proliferative effects in cancer cells, and favorable ADME/T characteristics (53, 16). Continuous structure–activity relationship efforts leveraging docking, QSAR, and contour-guided design are thus essential to realize improved anticancer phthalazine-based PARP1 inhibitors.

Comparative contour map analysis of active and inactive molecules across steric, hydrophobic, electrostatic, hydrogen bond acceptor (HBA), and hydrogen bond donor (HBD) fields provides key insights into structure–activity relationships. The relative contributions of the different physicochemical fields in the developed 3D-QSAR model were analyzed to better understand the structural features governing the biological activity of the studied compounds. The analysis revealed that steric interactions were the most influential factor, contributing 35.6% to the overall model. This indicates that the spatial arrangement and size of substituents around the molecular scaffold play a major role in determining the binding affinity and activity of the compounds within the target binding pocket. The hydrogen bond acceptor field was the second most significant contributor, accounting for 25.0% of the model. This suggests that the presence and positioning of functional groups capable of accepting hydrogen bonds are important for favorable interactions with amino acid residues in the active site. In addition, hydrophobic interactions contributed 19.5%, highlighting the importance of hydrophobic contacts between the ligands and non-polar regions of the receptor.

In contrast, electrostatic interactions contributed 10.2%, while hydrogen bond donor effects accounted for 9.7%, indicating a comparatively smaller influence on overall activity. Although these interactions are less dominant, they may still play supportive roles in stabilizing ligand–receptor binding. Overall, the field contribution analysis suggests that steric complementarity, hydrogen bond-accepting capability, and hydrophobic interactions are key determinants of the biological activity of the investigated compounds.

The steric contour maps (Figure 1) illustrate how the spatial arrangement and bulkiness of substituents influence biological activity. In these maps, green contours represent regions where bulky substituents are favorable for activity, while yellow contours indicate areas where steric bulk is unfavorable. For the active molecule (Figure 1A), large green contours are observed around specific regions of the molecular framework, particularly near the succinimide, which enhances receptor binding. These regions suggest that the receptor pocket can accommodate additional bulk at these positions, allowing favorable hydrophobic or van der Waals interactions. In contrast, yellow contours are positioned near areas where steric congestion would interfere with receptor binding or disrupt optimal molecular orientation. The distribution of these contours in the active molecule, above the succinimide group, indicates a well-balanced steric environment that fits effectively within the receptor site. In the inactive molecule (Figure 1B), the pattern is notably different. The green (favorable) region is located around the NH groups of the alkyl chain, suggesting less efficient occupation of the receptor pocket. Meanwhile, the yellow (unfavorable) contours often overlap with the alkyl chain present in the inactive compound, indicating steric clashes or restricted accommodation within the binding site that are absent in the active molecule.

The hydrophobic contour maps (Figure 2) generated from the 3D-QSAR analysis provide valuable insight into how hydrophobic interactions influence the biological activity of the studied compounds. The yellow and blue contour regions represent areas where hydrophobic and hydrophilic (or less hydrophobic) substituents, respectively, are favorable for enhanced activity. A comparative analysis of the active and inactive molecules reveals distinct differences in how these regions are occupied, helping to explain their contrasting activity profiles.

In the active molecule (Figure 2A), the major yellow contours are observed near the CH2 group of the succinimide group and around the central aromatic ring system. These regions indicate that hydrophobic substituents at these positions are beneficial for receptor binding and biological activity. The active molecule effectively occupies these hydrophobic-favored zones with nonpolar substituents, facilitating favorable van der Waals and hydrophobic contacts within the binding site.

In the inactive molecule (Figure 2B), the hydrophobic-favored zone is located around the alkyl chain and central benzene ring, showing alignment with these hydrophobic preference zones. The hydrophilic groups of the inactive compound extend toward the blue contour, likely the NH groups, indicating placement of polar substituents in regions where hydrophilic or polar interactions are preferred. Thus, with respect to hydrophobic effects, the inactive molecule also adequately occupies the yellow hydrophobic-favored pockets and blue hydrophilic regions observed in the active analog.

The electrostatic contour maps of the active and inactive molecules (Figure 3) show clear differences in charge distribution around important regions of the structures. In the active molecule (Figure 3A), the blue contour (positive effect) appears near the succinimide and central aromatic rings, suggesting that the presence of electron-donating or less electronegative groups in this area increases biological activity. The red contour (negative effect) is located around the carbonyl atoms, indicating that electron-withdrawing groups are favorable in this region for improving activity.

In contrast, the inactive molecule (Figure 3B) shows the blue contour near the amine and side-chain carbonyl groups, and the red contour close to the central aromatic ring. This opposite pattern suggests that the electron distribution in the inactive compound does not match the preferred electrostatic environment required for receptor binding, which may explain its reduced activity.

The electrostatic map indicates a bipolar requirement for activity: positive electrostatic potential in the vicinity of the succinimide and central aromatic ring (blue lobes) and negative electrostatic potential around the carbonyl/Cl region (red lobes). Functionally, this suggests that the ligand binds into a pocket that presents complementary negative character opposite the blue region and positive character opposite the red region. Thus, placing protonatable/basic groups to generate positive potential in the blue volumes and electronegative/hydrogen-bond-acceptor groups in the red volumes should increase receptor complementarity and potency.

The hydrogen bond acceptor contour maps (Figure 4) reveal how subtle variations in the position of acceptor features can strongly influence biological activity. In the active molecule (Figure 4A), the red (positive) contours are mainly located near the carbonyl group and the chlorine-substituted region of the aromatic ring. These areas indicate favorable zones for hydrogen bond acceptor groups, meaning that electron-rich atoms at these positions can effectively interact with hydrogen bond donor residues in the receptor binding site. Such interactions often stabilize the ligand–receptor complex and enhance activity. The presence of a magenta (negative) contour near the upper region of the succinimide moiety, however, indicates that additional acceptor character in that part would not be beneficial, likely due to steric hindrance or an unsuitable electrostatic environment in the receptor pocket. In contrast, the inactive molecule (Figure 4B) displays a different pattern. The positive (red) contours appear around the benzene ring and slightly near the alkyl chain, regions that are generally less accessible or less likely to form strong hydrogen bonds within a typical binding pocket. Meanwhile, the negative (magenta) contour located near the carbonyl group suggests that the acceptor feature in this position is unfavorable for binding. Because there is a carbonyl group in this region, this may lead to repulsive interactions or poor alignment with the receptor’s donor residues, weakening overall binding affinity.

The hydrogen bond donor contour maps (Figure 5) for both the active and inactive molecules show a similar blue-violet contour near the amine group of the fused aromatic ring, indicating that this region is favorable for hydrogen bond donor interactions. This suggests that the presence of a donor at this position is important for maintaining biological activity. However, despite having the same favorable region, the inactive molecule does not exhibit strong activity, which may be due to different groups in the unfavorable region (Figure 5B). A cyan contour, which represents an unfavorable region for hydrogen bond donors, is located near the succinimide carbonyl region in the active molecule and near the amine groups in the inactive molecule. In the active compound, this unfavorable region is near the carbonyl, which is an acceptor group, whereas in the inactive molecule the amine groups are donor groups. In the inactive molecule, the donor groups (NH groups) are positioned close to the cyan area, which could result in repulsive or geometrically unfavorable interactions, thereby reducing binding affinity.

The docking results provide compelling evidence that specific molecular features and interaction profiles are strongly correlated with enhanced binding affinity toward PARP1. Across the top-performing compounds, a recurring interaction pattern was observed: hydrogen bonding with GLY863 and SER904, π–π stacking with TYR907, and, in several cases, additional contacts with MET890 and GLN759. These interactions appear to define a pharmacophoric core essential for high-affinity binding.

Interestingly, while the co-crystallized ligand fluzoparib relies heavily on its conserved H-bonds with GLY863 and SER904, some of the discovered compounds (e.g., Compound 42 and Compound 63) not only preserved these interactions but also introduced additional stabilizing features. For instance, multiple π–π interactions (absent or limited in the intrinsic ligands) were consistently observed in the designed molecules, suggesting that these aromatic stacking contacts with TYR907 and TYR896 provide a crucial energetic advantage.

A closer comparison between Compounds 6, 9, and 13 (Table 3), which show both high docking scores and predicted activities, reveals that functional groups capable of dual hydrogen bonding (e.g., amide, urea, and carbonyl linkers) often lead to enhanced network formation within the binding site. This implies that, beyond targeting specific residues, the spatial orientation and multiplicity of donor/acceptor groups are key to maximizing interactions. Additionally, ligand flexibility may play a role; compounds that can adapt to engage multiple binding pockets or side chains (e.g., Compound 9 with four hydrogen bonds and π–π stacking) appear to benefit from entropic compensation and tighter binding.

Importantly, the docking results also highlight the differential contributions of non-covalent interactions. For example, Compound 18, despite forming the key H-bond pair, exhibited a significantly lower docking score of -2.514, likely due to weaker secondary interactions or an unfavorable orientation within the binding cleft. This contrast emphasizes that simply reproducing conserved interactions is not sufficient; synergistic effects between hydrogen bonding and hydrophobic/aromatic contacts are necessary for optimal activity.

From a design standpoint, the findings indicate that increasing the number of interaction points (particularly hydrogen bonds and π–π stacking) may contribute to the development of stronger and more efficient ligands. The enhanced performance of Compounds 42 and 63 over the benchmark fluzoparib underscores the predictive value of combining docking with QSAR approaches: not only were better binders identified, but the predicted activities were also higher.

In sum, the docking outcomes validate the importance of conserved hydrogen bonding while also highlighting structural features and interactions that differentiate high-affinity binders, thereby providing a roadmap for rational design. Future analogs should aim to preserve the GLY863–SER904 H-bond motif while reinforcing π–π stacking with TYR907 and enhancing peripheral contacts (e.g., with MET890 or GLN759) to further stabilize the ligand–protein complex.

The molecular dynamics simulations further validate the docking results by demonstrating the stability of the three selected ligand–PARP1 complexes over 100 ns. RMSD analysis of the protein backbone (Figure S4) showed deviations below 0.3 nm, confirming that none of the ligands induced major structural rearrangements of the binding site and that the receptor remained conformationally stable throughout the trajectory. This is in agreement with docking predictions, in which all three ligands occupied the canonical PARP1 active site.

Ligand RMSD analysis (Figure S5) revealed that CMP1 and CMP63 maintained relatively low deviations, suggesting stable binding conformations, whereas CMP42 displayed somewhat higher fluctuations. Closer inspection showed that this deviation arose from flexible domains of CMP42 outside the active site rather than from unbinding, which explains why this compound still maintained high binding affinity as predicted in docking. RMSF analysis (Figure 8) supported these findings, showing higher fluctuations for CMP42 (0 - 0.4 nm) compared with CMP1 and CMP63 (0 - 0.2 nm), consistent with its partially extended binding orientation.

Hydrogen bond occupancy analysis (Figure 9) highlighted Ser904 as the key anchoring residue across all complexes, with occupancies of 89%, 97%, and 92% for CMP1, CMP42, and CMP63, respectively. This is fully consistent with docking, where Ser904 and Gly863 were predicted as crucial interaction hotspots. Interestingly, CMP42 not only strengthened its dominant hydrogen bond with Ser904 but also engaged Arg865 (44%), exploiting a positively charged pocket not significantly utilized by CMP1 or CMP63. CMP63, in turn, displayed a dual anchoring mode via strong hydrogen bonding to both Ser904 (92%) and Gln759 (59%), stabilizing the ligand in the binding cavity and reflecting its favorable docking score and predicted activity. CMP1 exhibited a more distributed interaction network, forming weaker additional hydrogen bonds with Gln717, Gln759, and His862, which may explain its slightly lower docking score compared with CMP42 and CMP63.

Overall, MD simulations reinforce the docking observations: conserved interactions with Ser904 and Gly863 are essential for ligand stabilization, while additional contacts such as Arg865 (CMP42) or Gln759 (CMP63) provide compound-specific enhancements. The strong agreement between docking predictions and MD trajectories supports the reliability of the QSAR-guided design and highlights CMP1 and CMP42 as the most promising PARP1 inhibitors among the studied molecules.

### 5.1. Conclusions

In this study, a field-based 3D-QSAR model was successfully developed for a series of phthalazine derivatives as PARP1 inhibitors, achieving robust statistical validation and providing meaningful contour maps that guided the design of new analogues. Docking studies confirmed the importance of conserved hydrogen bond interactions with key residues Ser904 and Gly863, as well as π–π stacking with Tyr907, in stabilizing ligand binding within the PARP1 active site. Several compounds, particularly CMP63 and CMP42, demonstrated improved docking scores and interaction profiles compared with intrinsic PARP1 inhibitors. Molecular dynamics simulations over 100 ns further validated these results by confirming complex stability, with conserved hydrogen bond occupancy and additional ligand-specific contacts such as Arg865 (CMP42) and Gln865 (CMP63) contributing to enhanced binding affinity. Overall, the combined QSAR, docking, and MD approaches provide strong evidence for the potential of phthalazine derivatives as novel PARP1 inhibitors, highlighting promising candidates for further experimental validation in anticancer drug discovery. The graphical abstract summarizes the discovery workflow of phthalazine derivatives as novel PARP1 inhibitors using 3D-QSAR modeling, molecular docking, and molecular dynamics simulations (Figure 10).

ijpr-25-1-169970-s001.pdf

## Data Availability

The data presented in this study are uploaded during submission as a supplementary file and are openly available for readers upon request.
