# Supplementary material for: Discovery of Phthalazine Derivatives as New PARP1 Inhibitors Through 3D-QSAR, Molecular Docking, and MD Studies
Source: Iran J Pharm Res. 2026 Jun 16;25(1):e169970. doi: 10.5812/ijpr-169970 (PMC13769048; doi:10.5812/ijpr-169970)
Supplement: Supplementary file 1 [file ijpr-25-1-169970-s001.pdf]

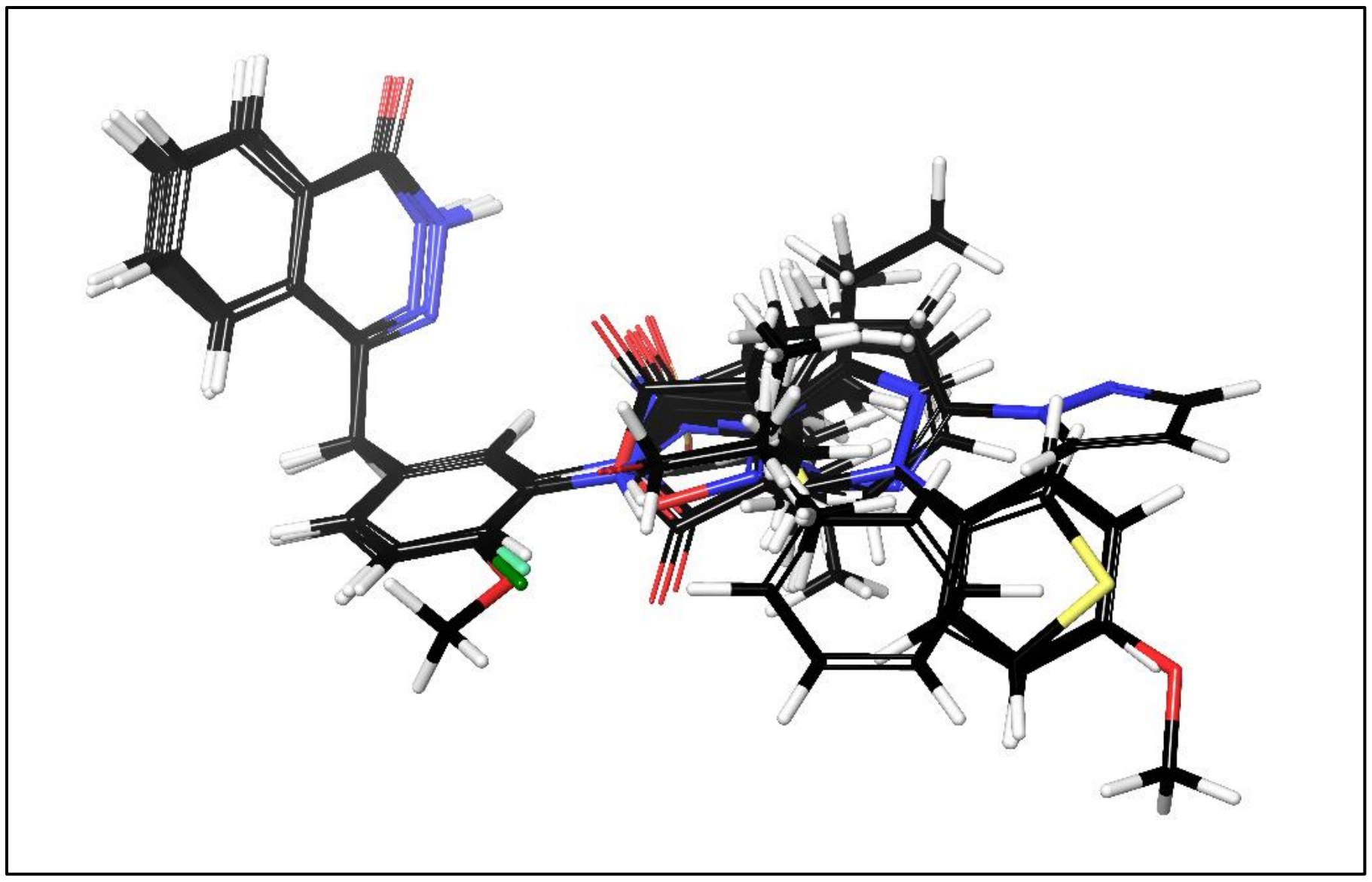

Fig. S1. The aligned phthalazine derivatives.

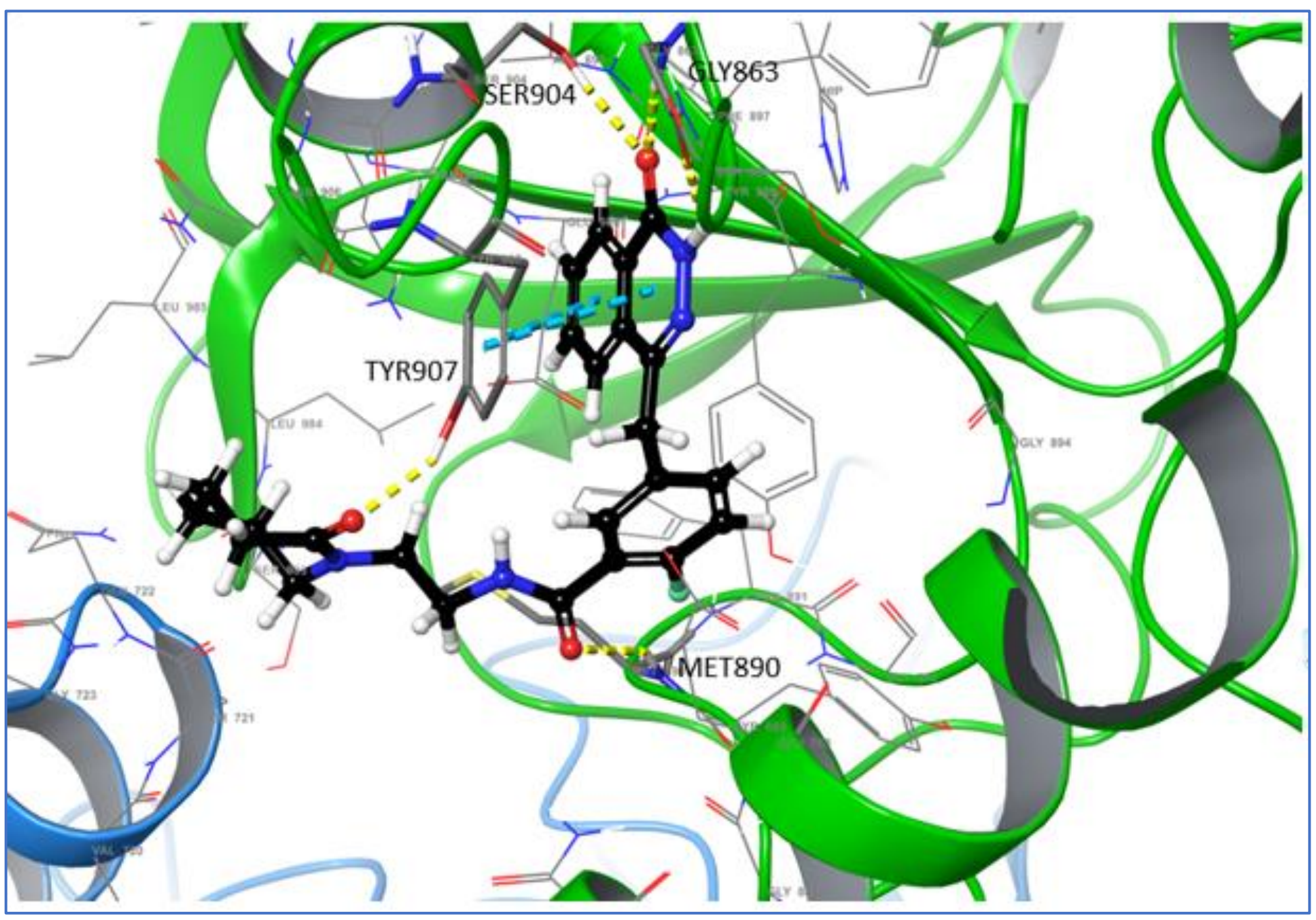

Fig.S2 . The presence of compound 9 among different residues of PARP1 and the 2D structures. Yellow dashed line indicates the hydrogen bond and blue dashed lines are Pi interactions in 3D illustration

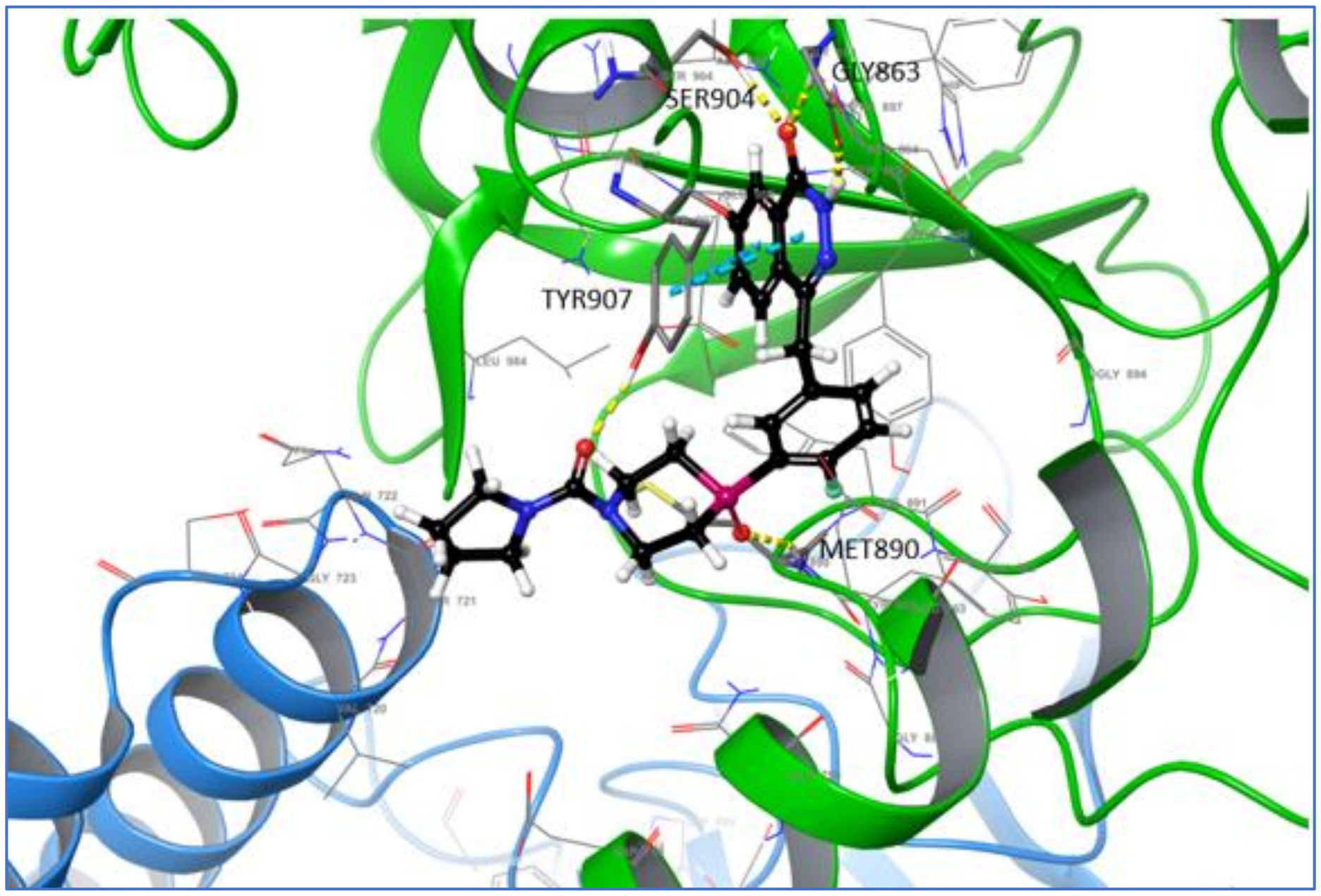

Fig.S3 . The presence of compound 13 among different residues of PARP1 and the 2D structures. Yellow dashed line indicates the hydrogen bond and blue dashed lines are Pi interactions in 3D illustration.

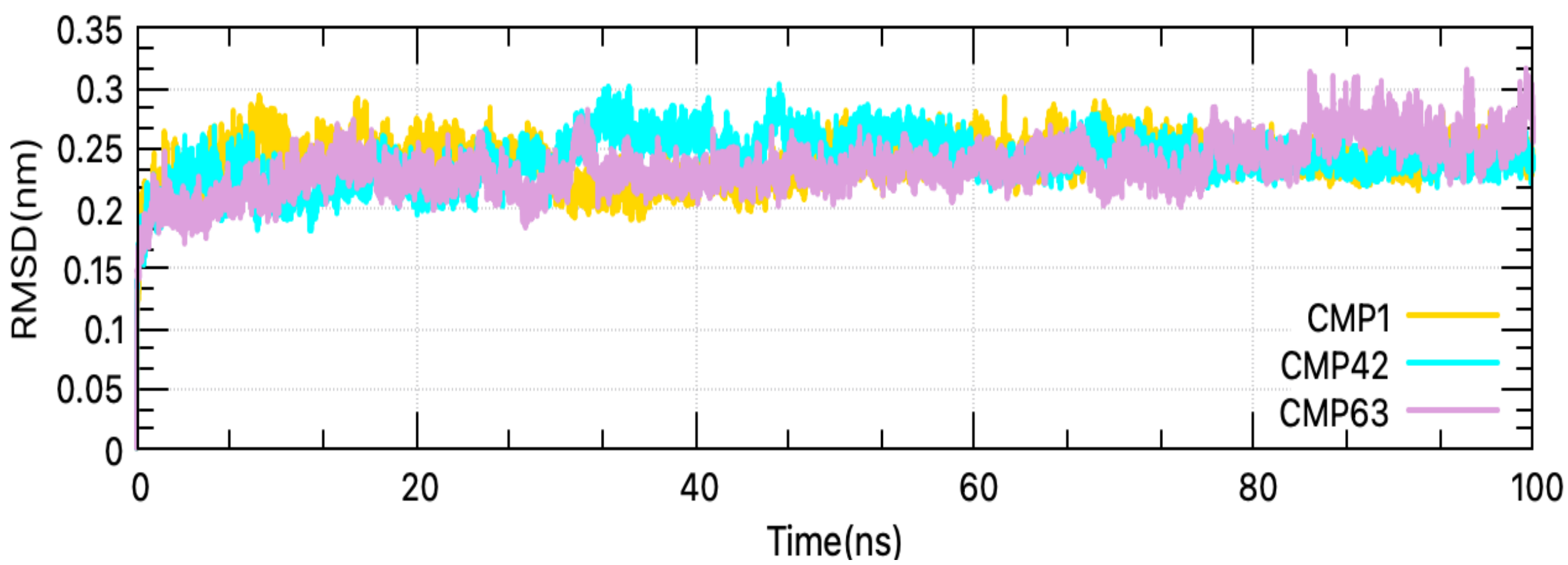

Fig. S4**.** RMSD analysis of the receptor over the 100-ns MD simulations. The plot illustrates the evolution of structural deviations relative to the initial conformation, providing insights into the global stability and conformational dynamics of the receptor in the presence of different ligands.

The consistently low RMSD values suggests that the ligand binding does not destabilize the protein.

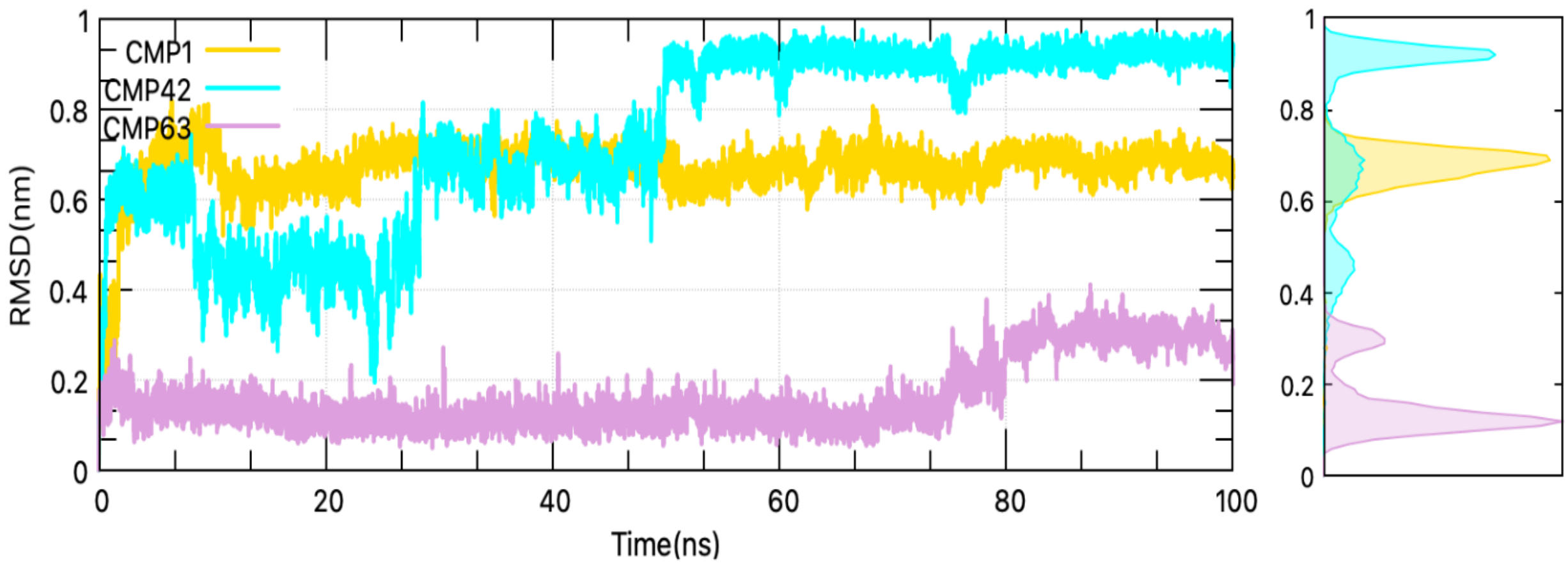

Fig. S5**.** RMSD analysis of the compounds during the 100-ns MD simulations, illustrating the structural stability and conformational fluctuations of each ligand within the binding site. The left panel shows the RMSD trajectories for CMP1, CMP42, and CMP63, while the right panel presents the corresponding RMSD distribution plots, highlighting the range and frequency of deviations. CMP1 and CMP63 exhibit relatively stable RMSD profiles with narrow distributions, indicating consistent binding poses, whereas CMP42 shows increased fluctuations, reflecting greater flexibility in certain ligand domains.

Table S1. The IC_{50} values and corresponding pIC_{50} activities of phthalazine derivatives reported in various studies

SMILE	Phthalazin derivatives	IC50 (μM)	pIC50 = -log (IC50)	QSAR set
CC(C)C(=O)Nc1cccc(Cc2nnc(O)c3ccccc23)c1	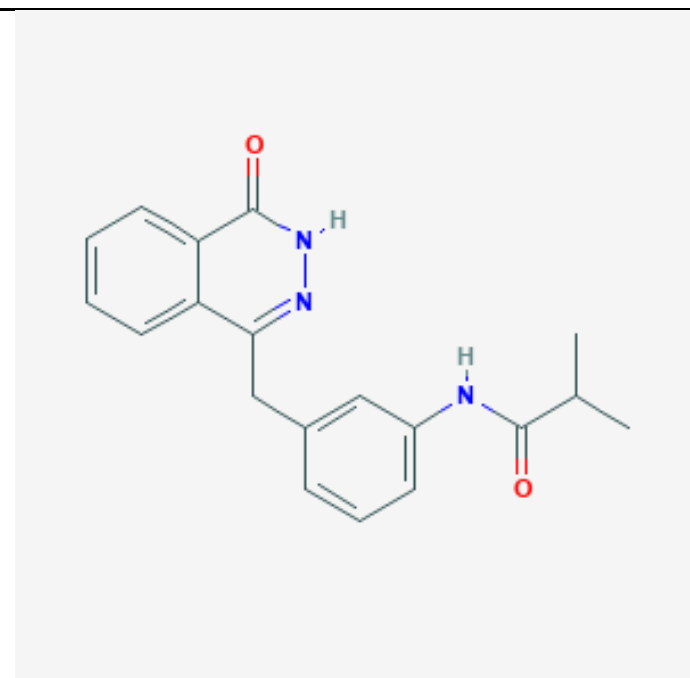	0.37	6.431	training
O=C1CCCN1c1cccc(Cc2n[nH]c(=O)c3ccccc23)c1	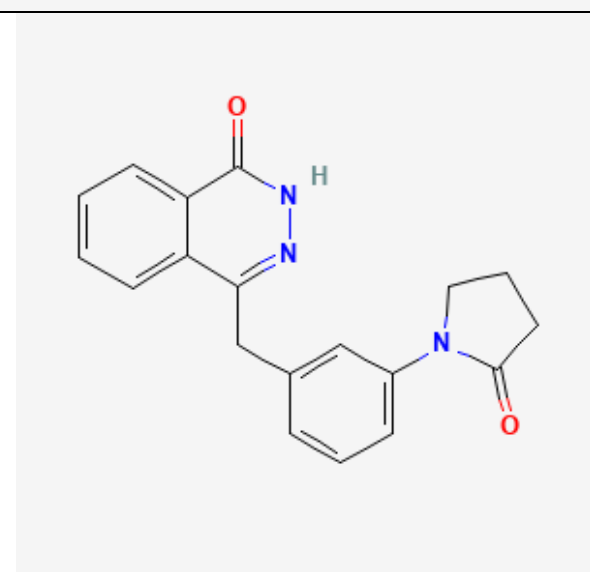	0.12	6.921	training
COc3ccc(Cc1nncc2ccccc12)cc3N4C(=O)CCC4=O	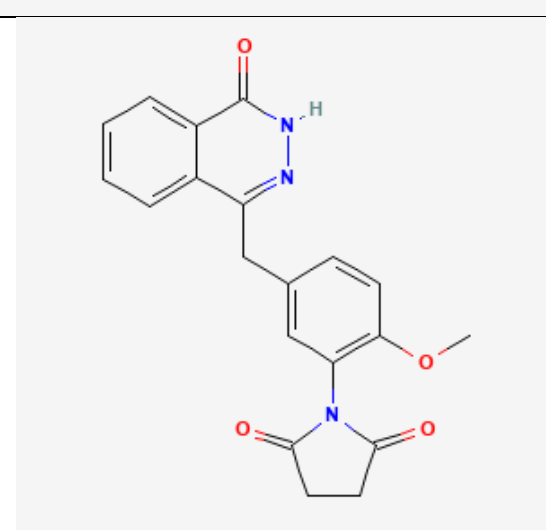	0.033	7.481	test
CN4CC(=O)N(c3cc(Cc1nncc2ccccc12)ccc3F)C(=O)C4	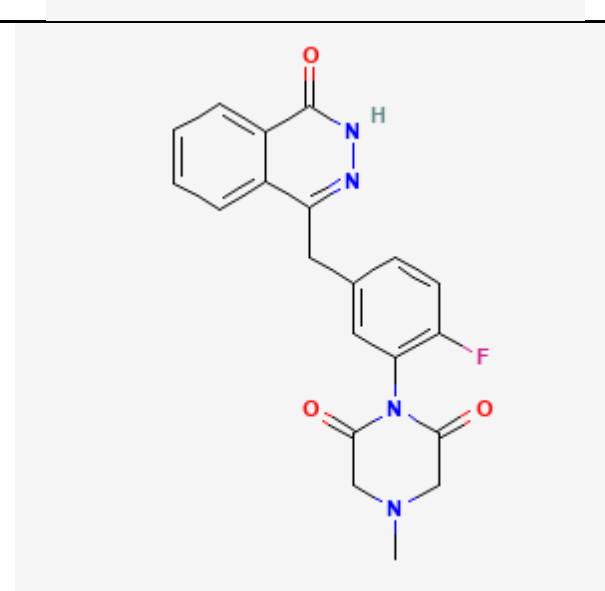	0.0095	8.022	test

O=C1CCC(=O)N1c4cccc(Cc2n[nH+]cc3ccccc23)c4	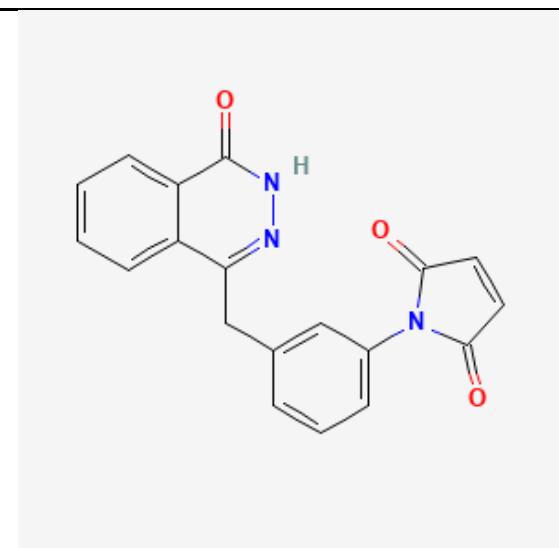	0.013	7.886	training
O=C1NN=C(C2=CC=CC=C12)CC3=CC(=CC=C3)NC(=O)C4CC4	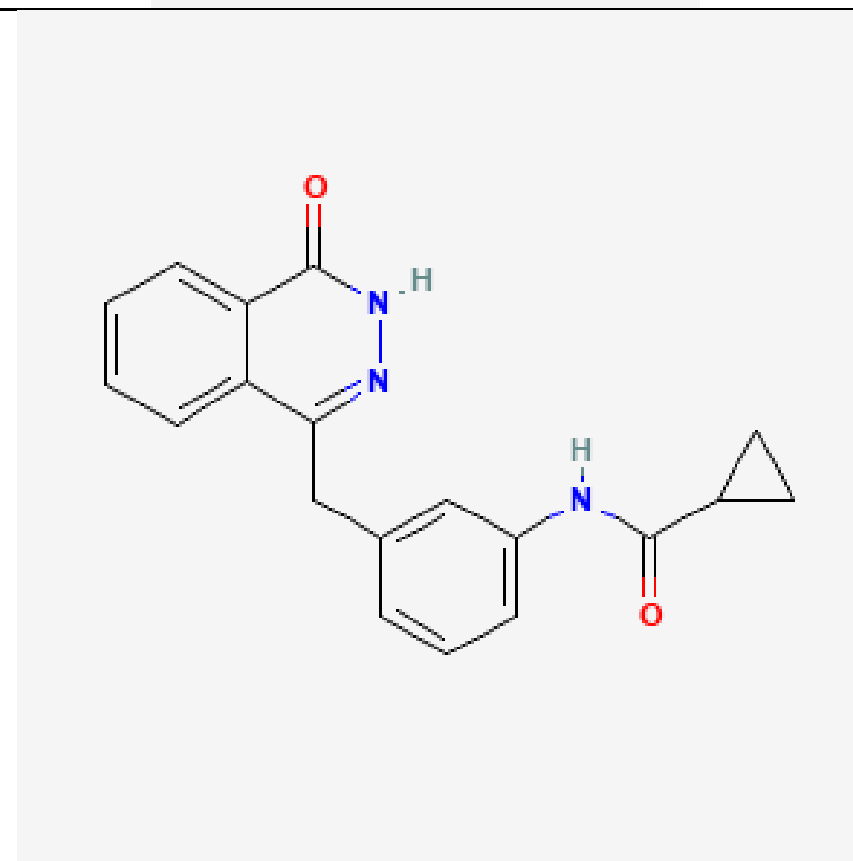	0.055	7.259	training
CC(=O)OC1=CC=CC(=C1)CC2=NN=C3C=CC=CC3C2=O	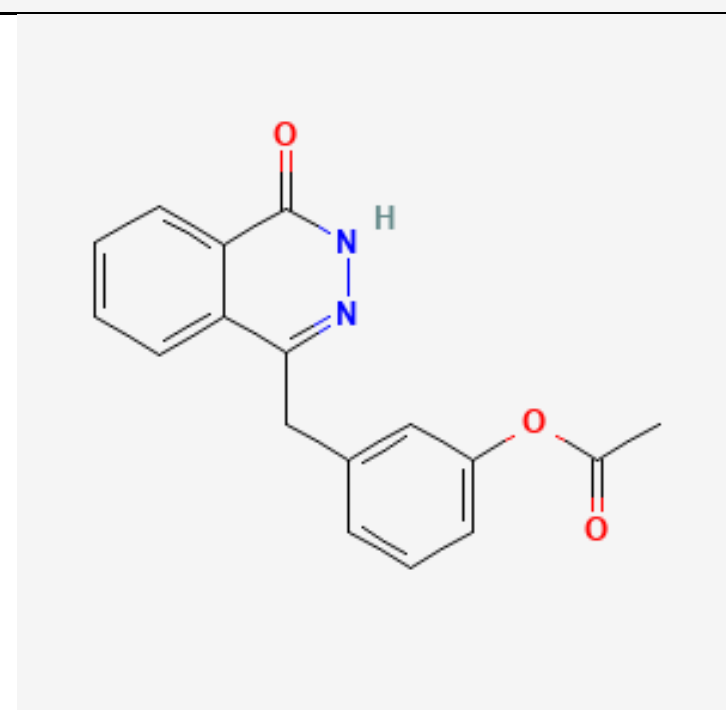	0.036	7.443	training
O=C1CCC(=O)N1C2=CC=CC(=C2)CC3=NN=CC4=CC=CC=C43	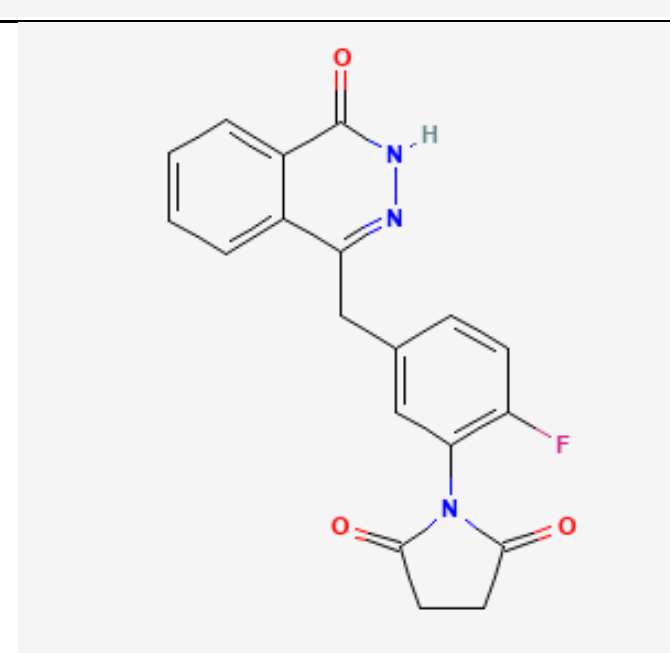	0.005	8.301	test

O=C1CCC(=O)N1C2=CC=CC(=C2)CC3=NN=CC4=CC=CC=C43	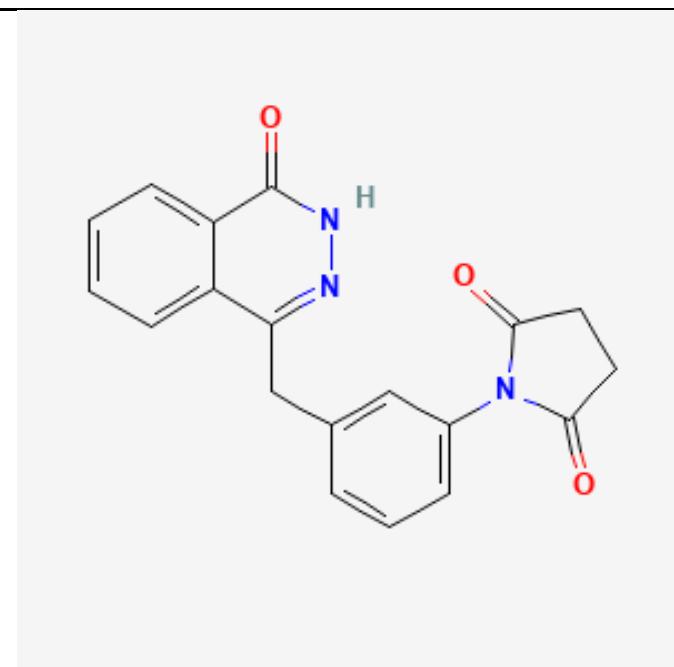	0.012	7.921	training
O=[c+]3nnc(Cc1ccccc1)c2ccccc23	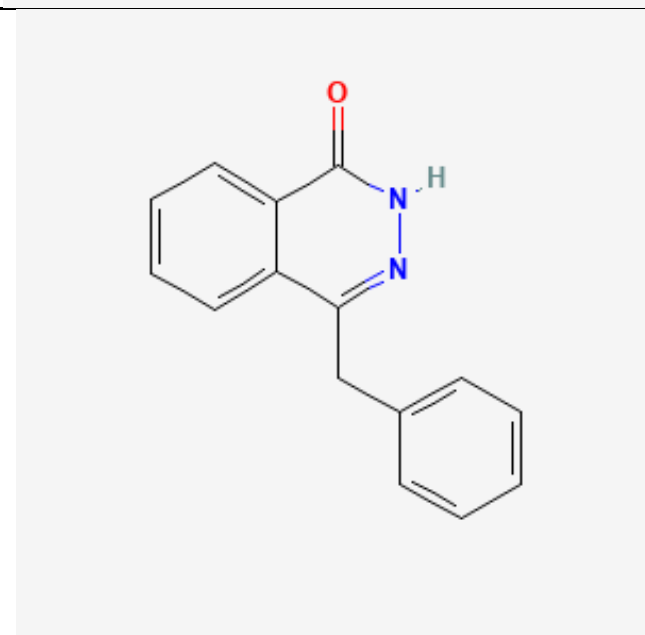	0.77	6.113	test
CC1NOC(N1)C2=CC=CC(C3C(=O)NN=C4C=CC=CC34)=C2	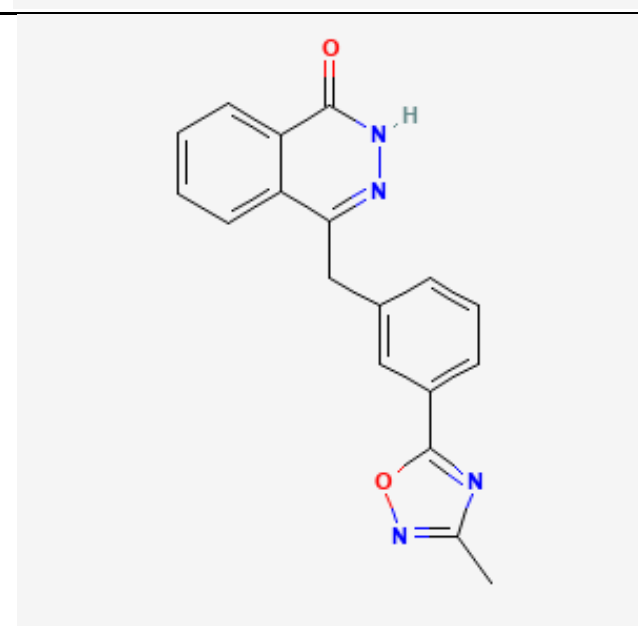	0.19	6.721	training
O=C1NCC(=O)C1C2=CC(=C(C=C2)CN3C=CC4=CC=CC=C4N3=O)Cl	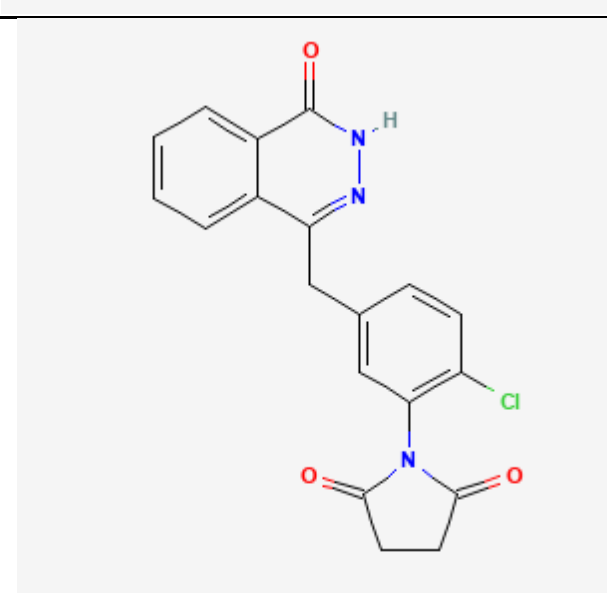	0.019	7.721	test
Cc1c(sc(n1)c2ccccc2)C(=O)Nc3cccc(c3)CN4C=CC5=CC=CC=C5N4=O	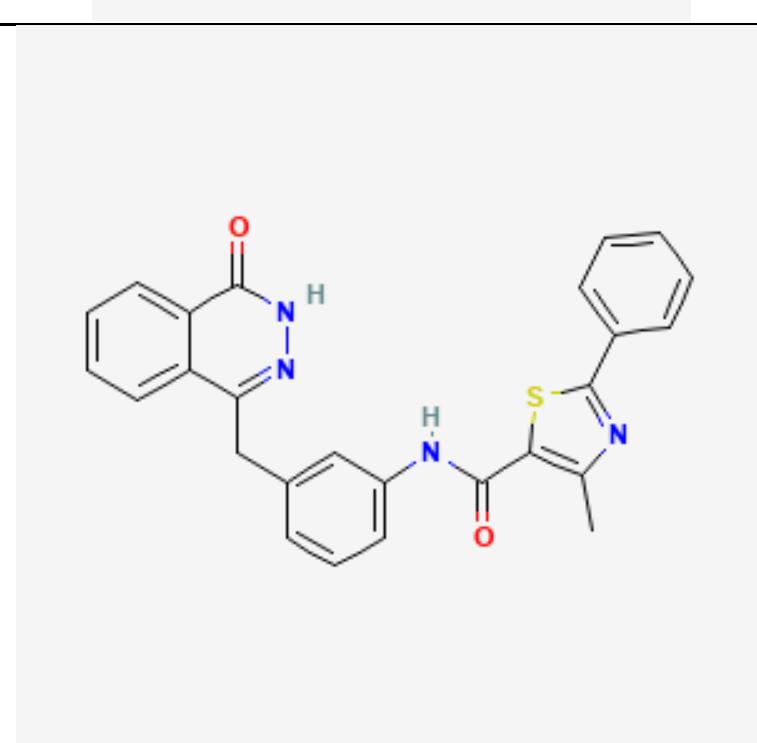	0.027	7.569	training

CCC(=O)Nc1cccc(c1)CN2C=CC3=CC=CC=C3C2=O	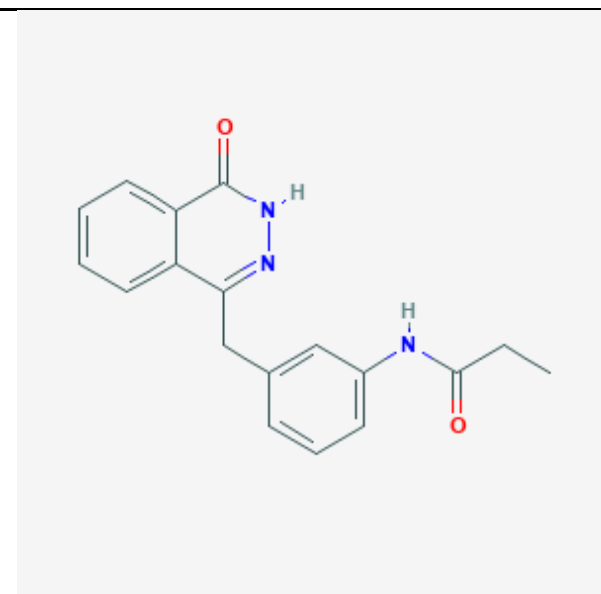	0.02	7.699	test
O=C(NC1=CC(=CC=C1)CN2N=NC3=CC=CC=C3C2=O)C4=CC=CS4C5=CS=CC5	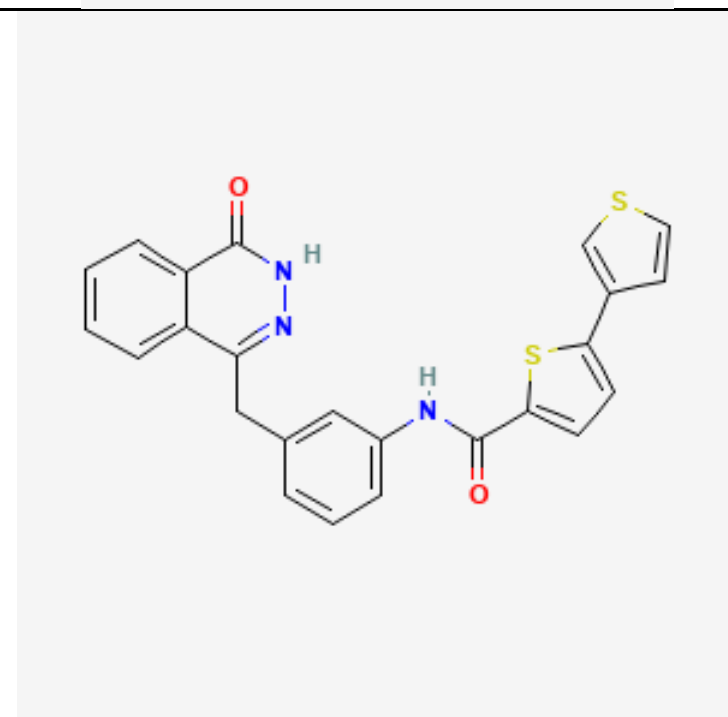	0.056	7.252	training
O=C2C1CC1C(=O)N2c5ccc(Cn4nnc3ccccc3c4=O)c(F)c5	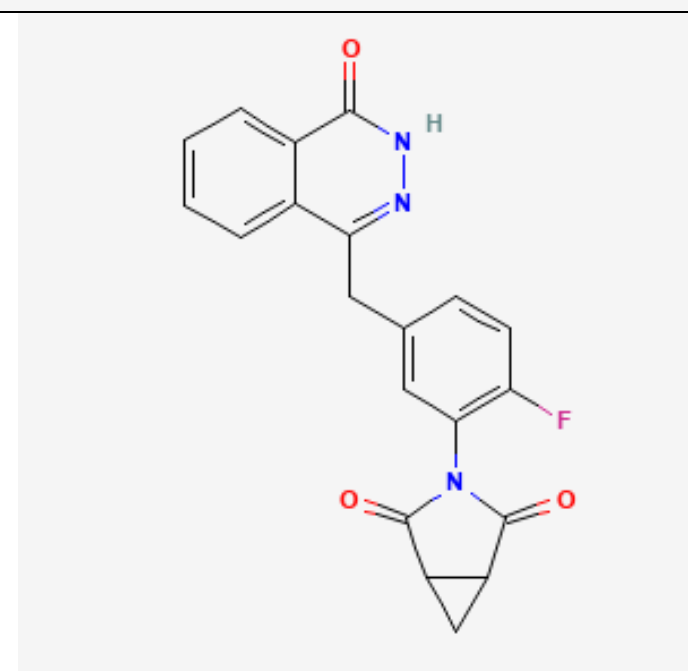	0.005	8.301	training
CCCCNC(=O)NC1=CC=CC(=C1)CN2N=NC3=CC=CC=C3C2=O	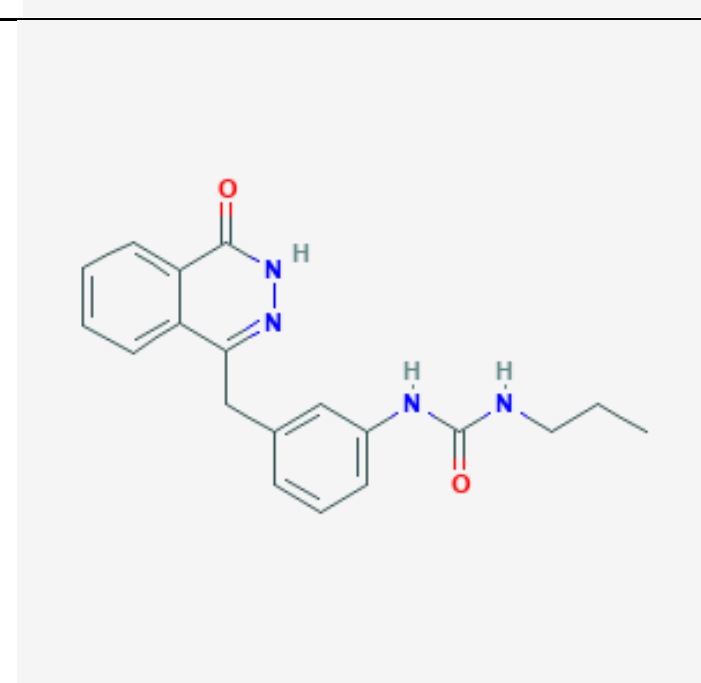	0.54	6.268	training
COC1=CC=C(C=C1)N2C=C(C(=N2)C)C(=O)NC3=CC=CC(=C3)CN4N=NC5=CC=CC=C5C4=O	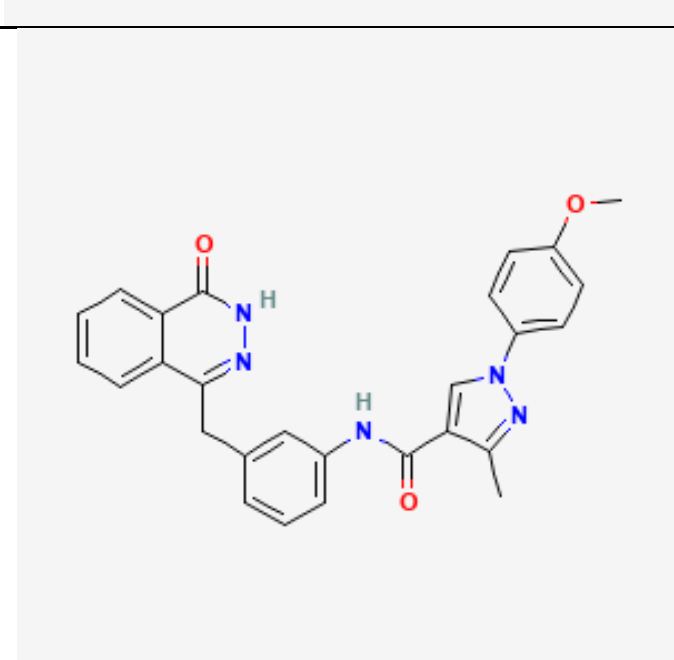	0.01	8	test

O=c2c1ccccc1c(=O)n2Cc3nn[c+](=O)c4ccccc34	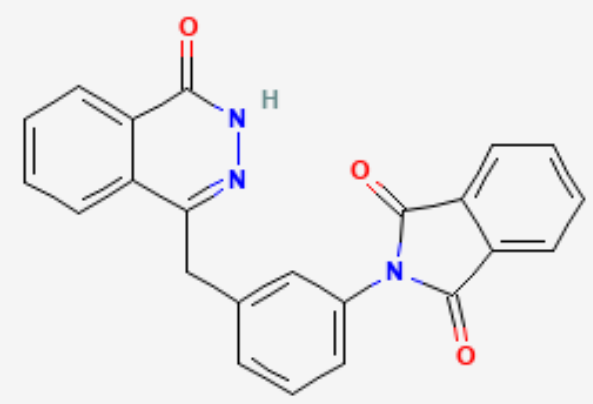	0.18	6.745	training
CC(=O)NC1=CC(=CC=C1)CC2=NN3C=CC=CC3C(=O)N2	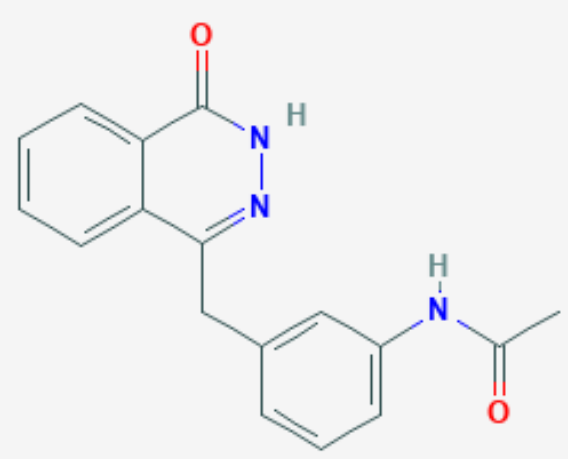	0.09	7.046	test
CCOC(=O)NC1=CC(=CC=C1)CC2=NN3C=CC=CC3C(=O)N2	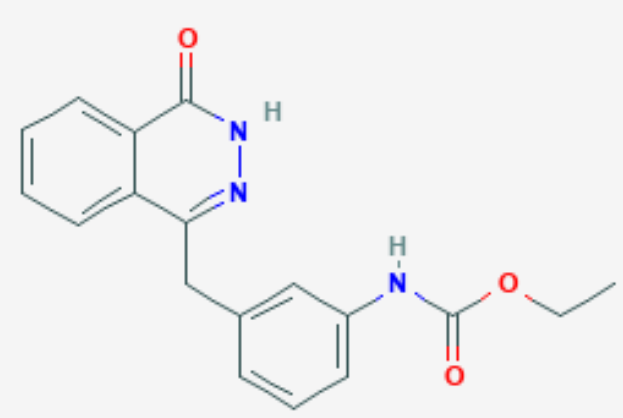	0.116	6.936	training
CCCC(=O)NC1=CC(=CC=C1)CC2=NN3C=CC=CC3C(=O)N2	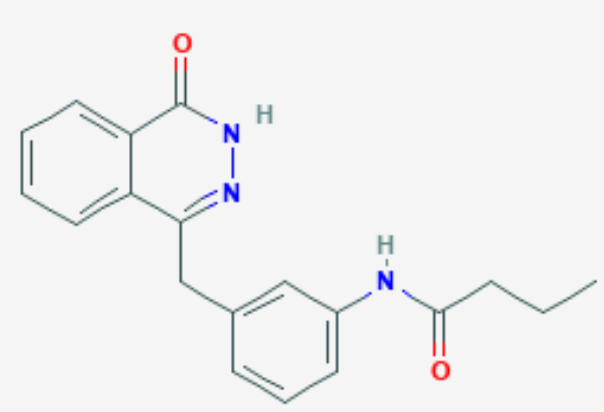	0.09	7.046	test

O=C(Nc3cccc(Cc1nn[c+](=O)c2ccccc12)c3)c5ccc(n4cccn4)cc5	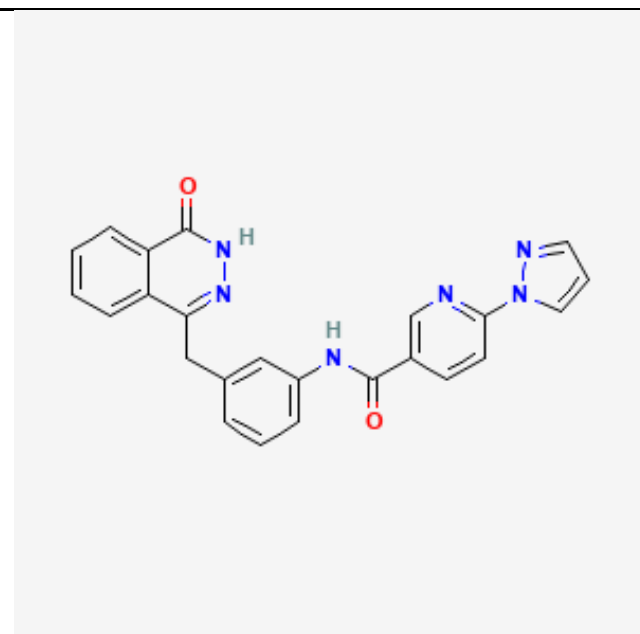	0.047	7.328	training
O=C1CCCC(=O)N1c4cc(Cc2nn[c+](=O)c3ccccc23)ccc4F	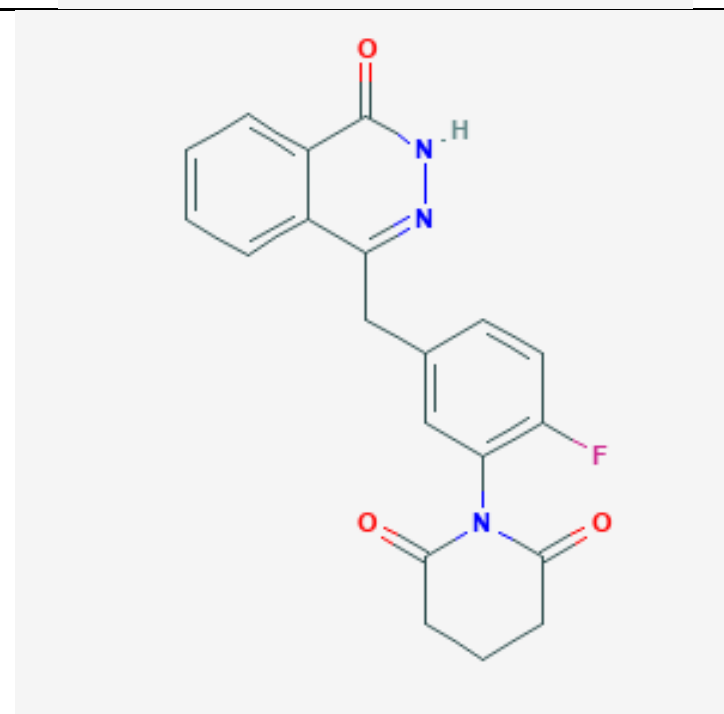	0.004	7.398	training
O=S(=O)(CC)NC1=CC=CC(=C1)CC2=NN3C=CC=CC3C2=O	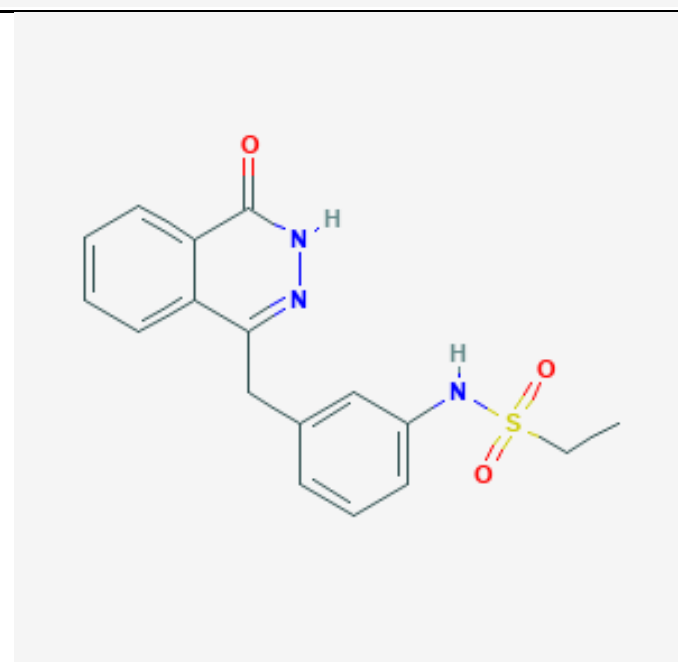	0.29	6.538	training
O=C2CC(c1ccccc1)C(=O)N2c5cc(Cc3nn[c+](=O)c4ccccc34)ccc5F	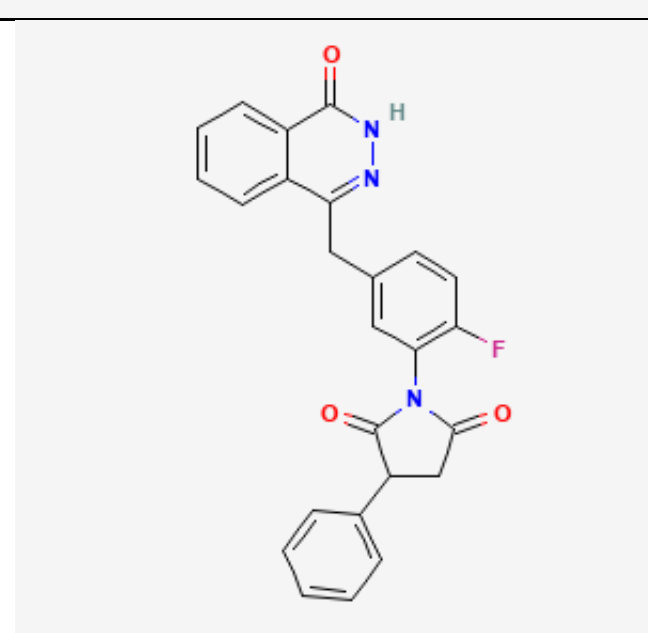	0.007	8.155	test
CC4(C)CC(=O)N(c3cc(Cc1nn[c+](=O)c2ccccc12)ccc3F)C4=O	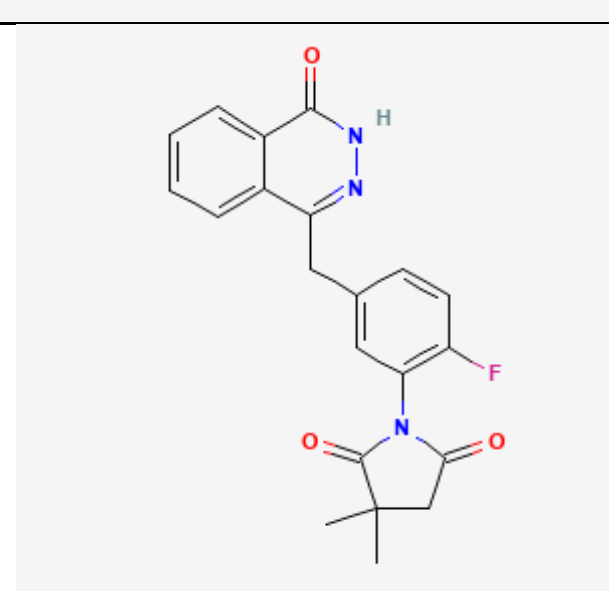	0.01	8.000	training

CCNC(=O)c3cccc(Cc1nn[c+](=O)c2ccccc12)c3	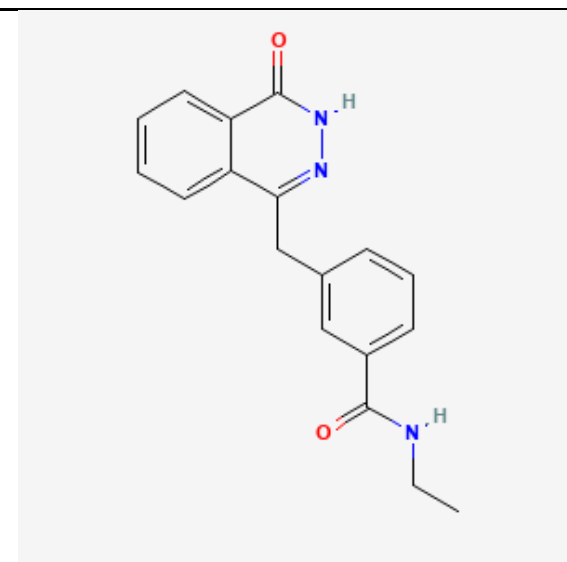	0.05	7.301	training
CC4CC(=O)C(c3cc(Cc1nn[c+](=O)c2ccccc12)ccc3F)C4=O	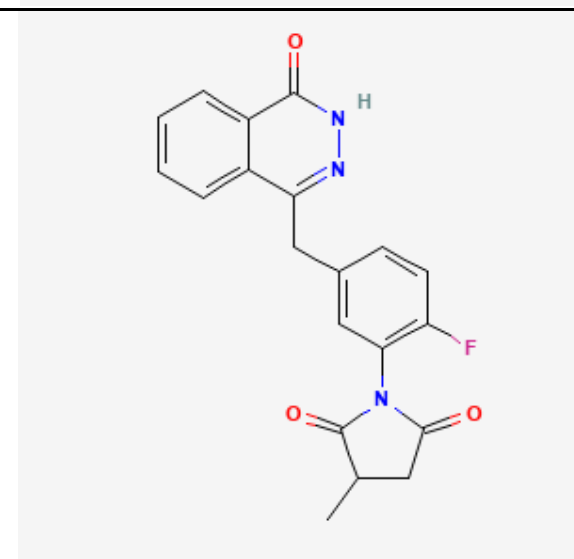	0.004	8.398	test
O=C(Nc3cccc(Cc1nn[c+](=O)c2ccccc12)c3)C(F)(F)F	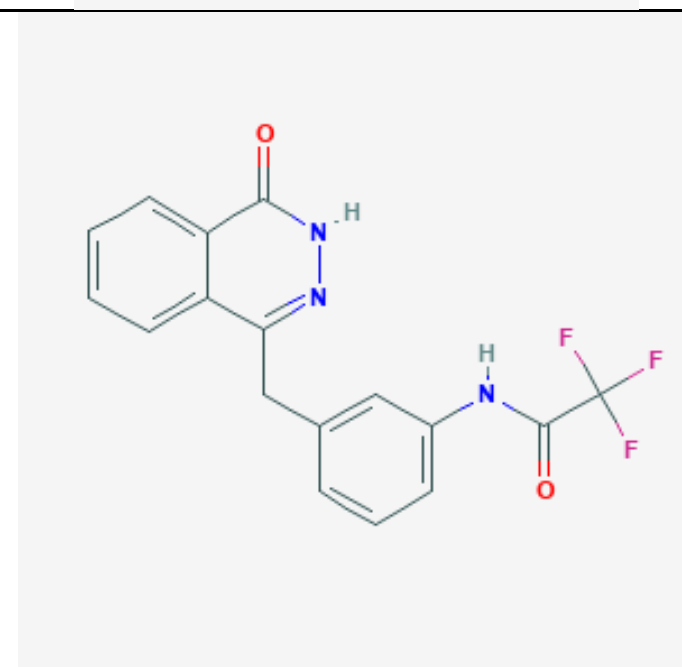	0.013	7.886	training

Table S2. The 2D structures and IC50 values of top 20 compounds obtained from docking

Name	2D structure and IC_{50}
Compound 1	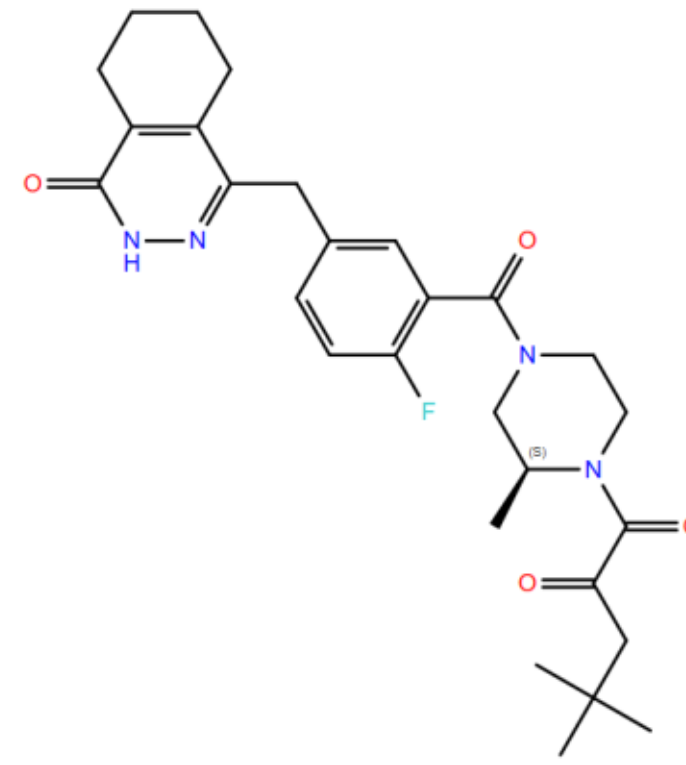 9.269
Compound2	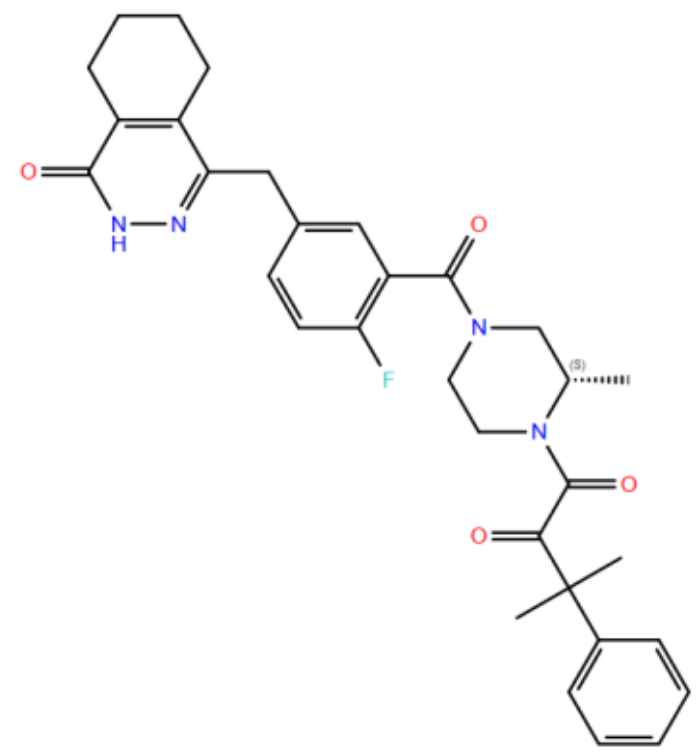 9.094
Compound3	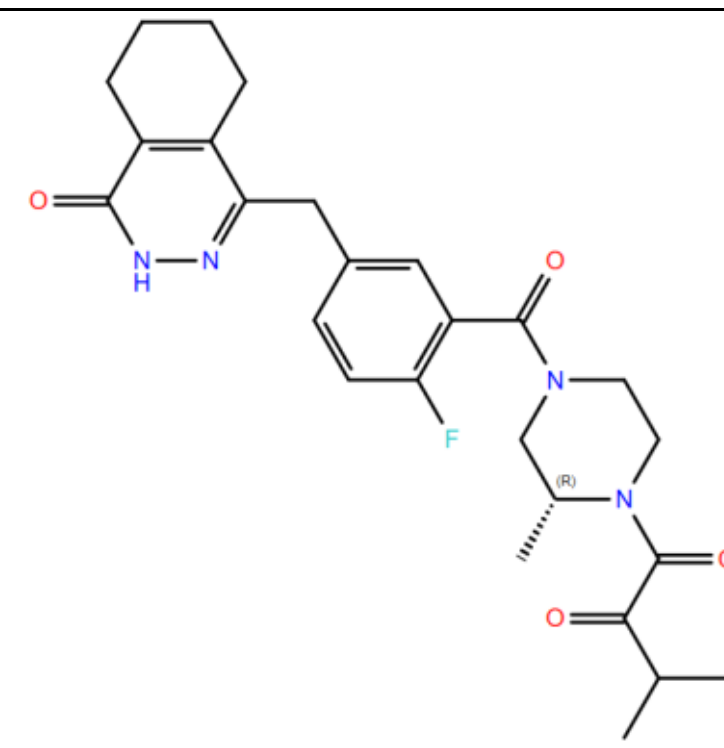 8.987
Compound5	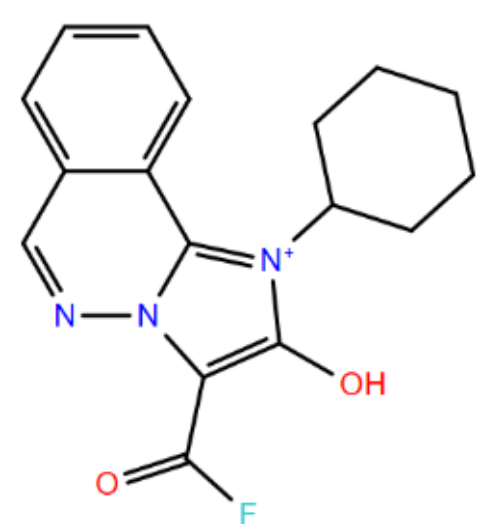 8.981
Compound6	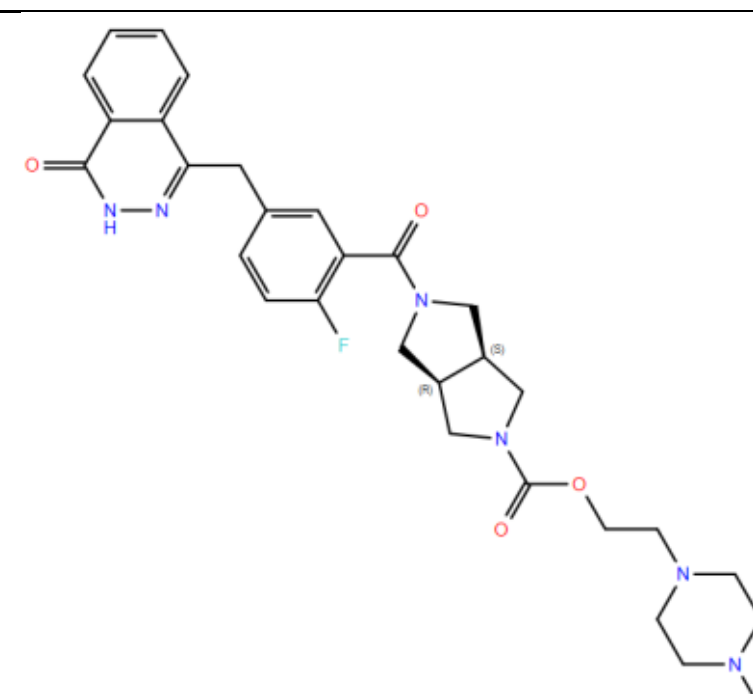 8.894

Compound7	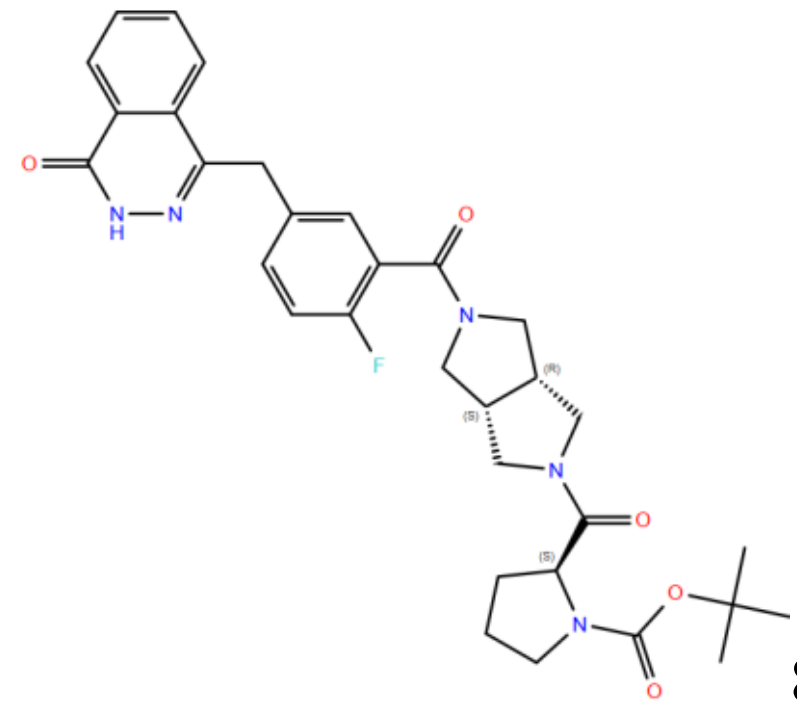 8.884
Compound8	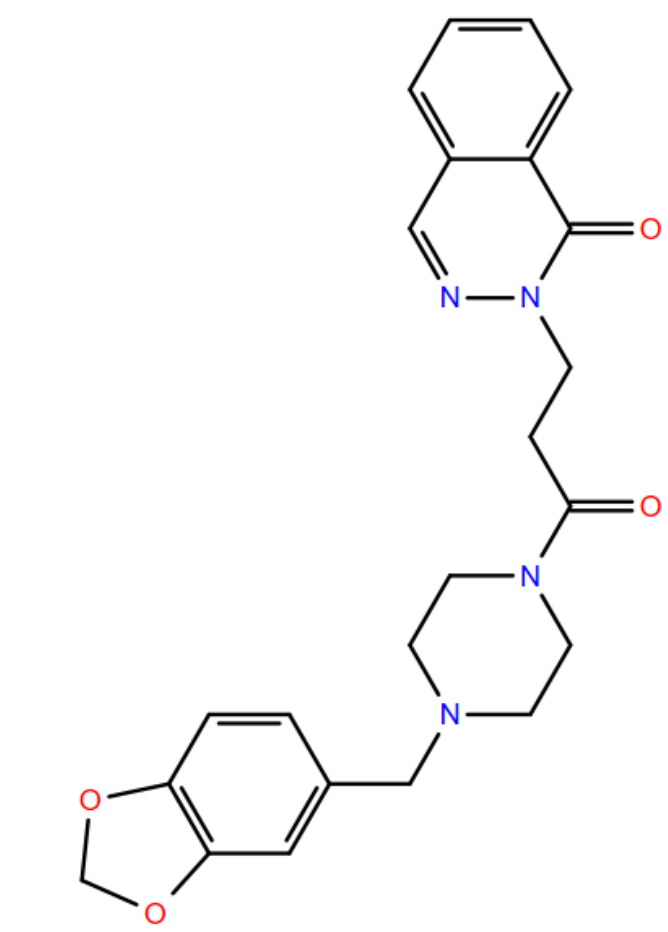 8.816
Compound9	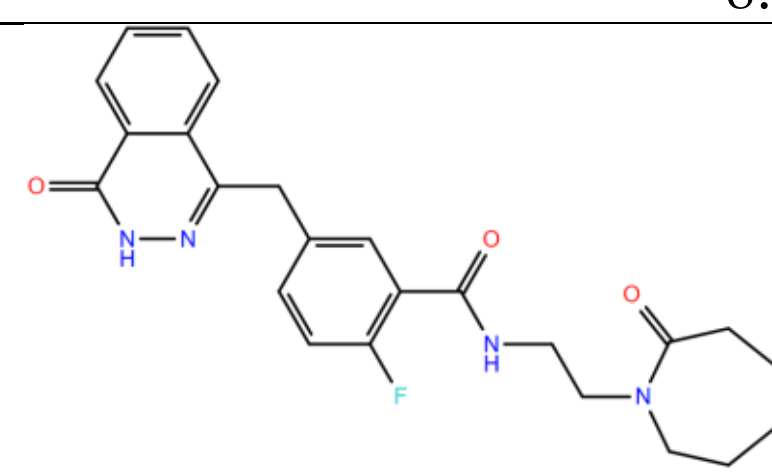 8.748
Compound10	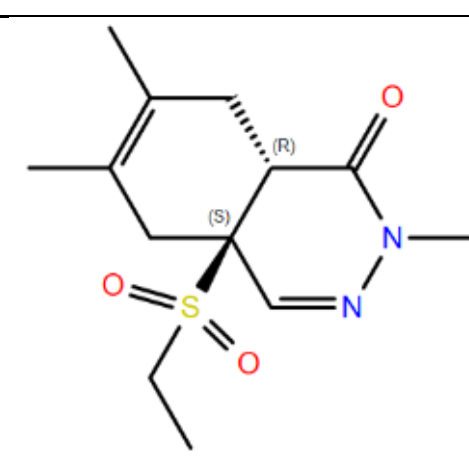 8.743
Compound12	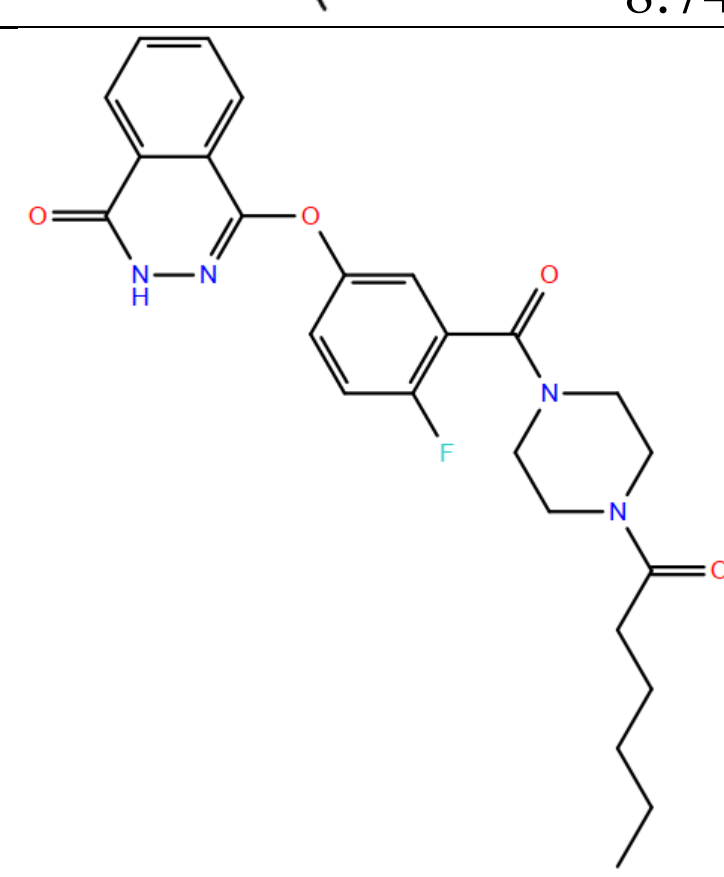 8.732

Compound13	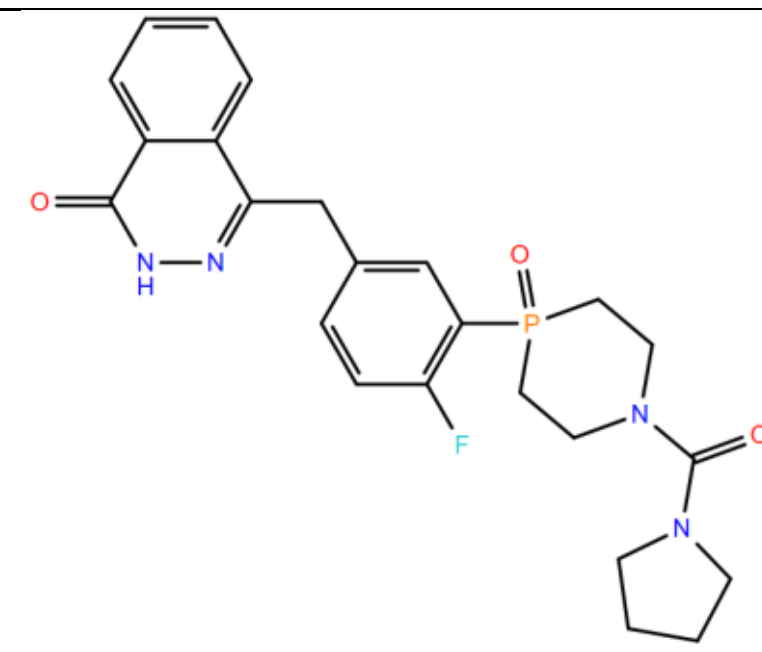 8.729
Compound14	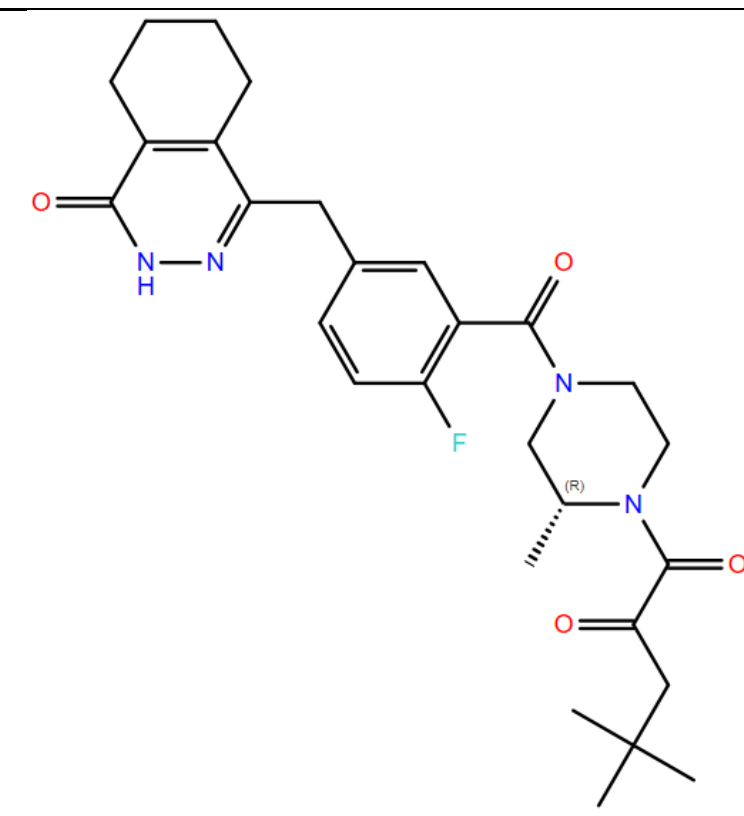 8.725
Compound15	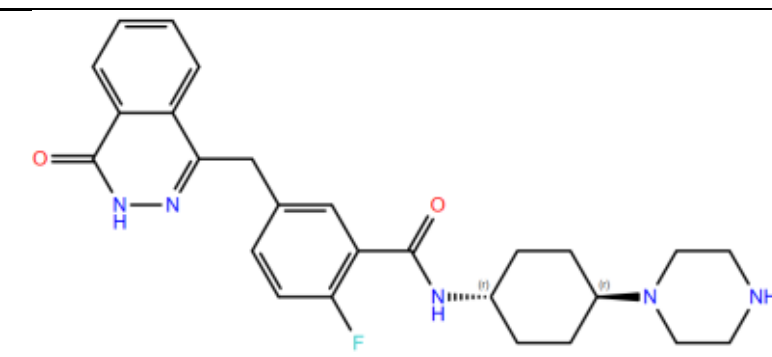 8.711
Compound16	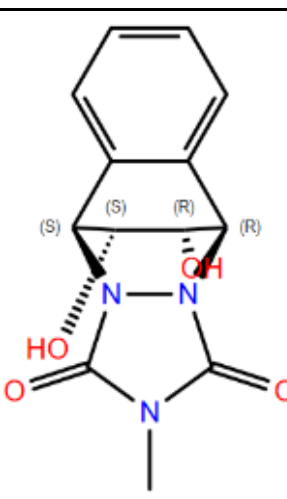 8.687
Compound17	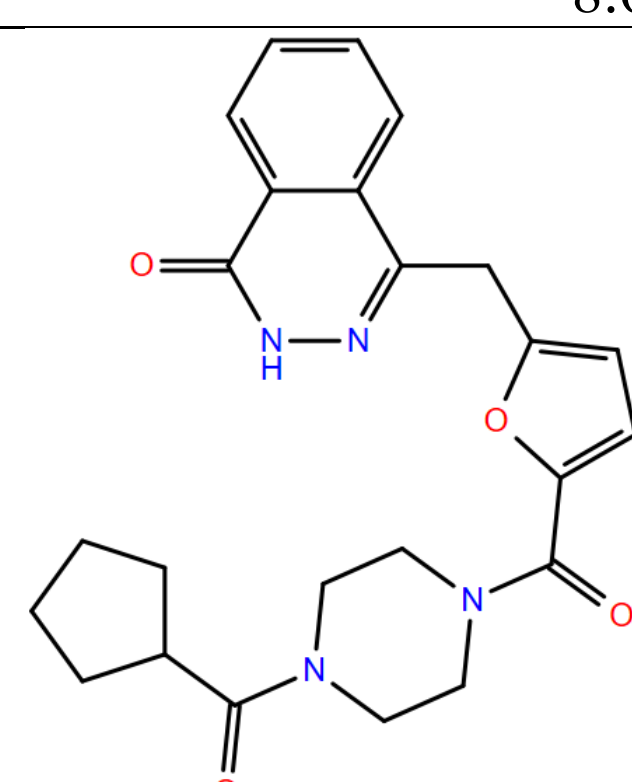 8.679

Compound18	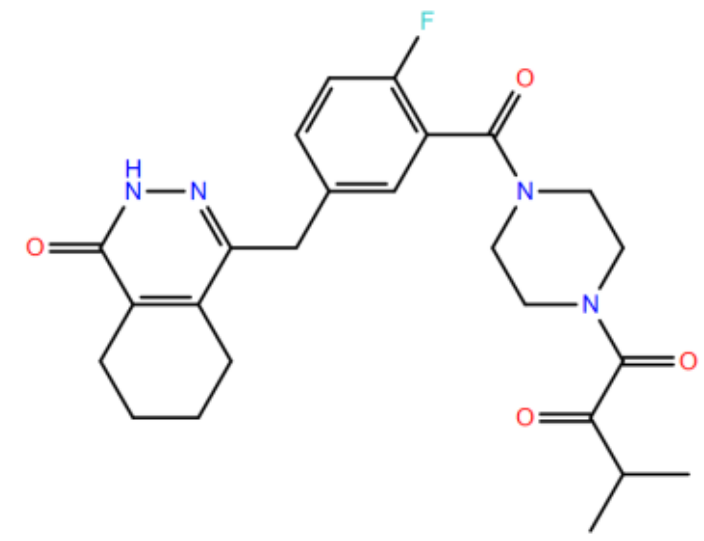 8.668
Compound19	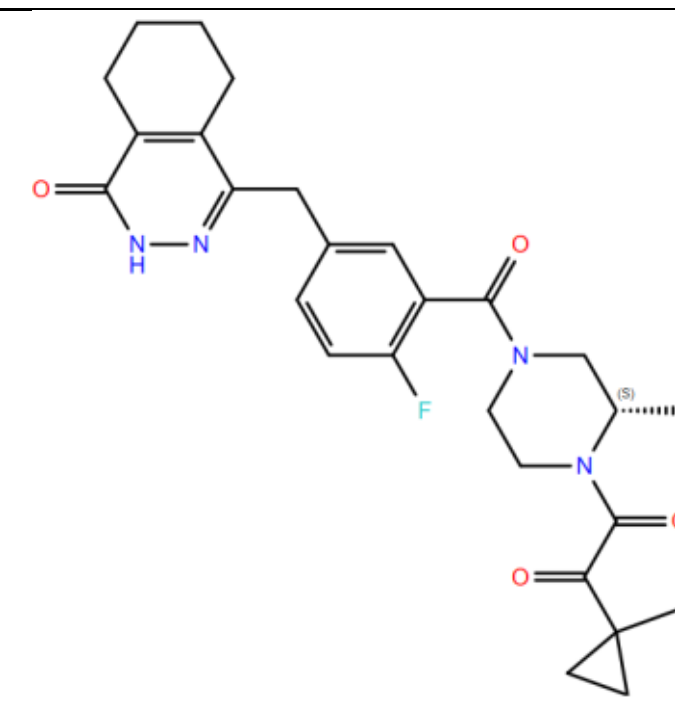 8.659
Compound20	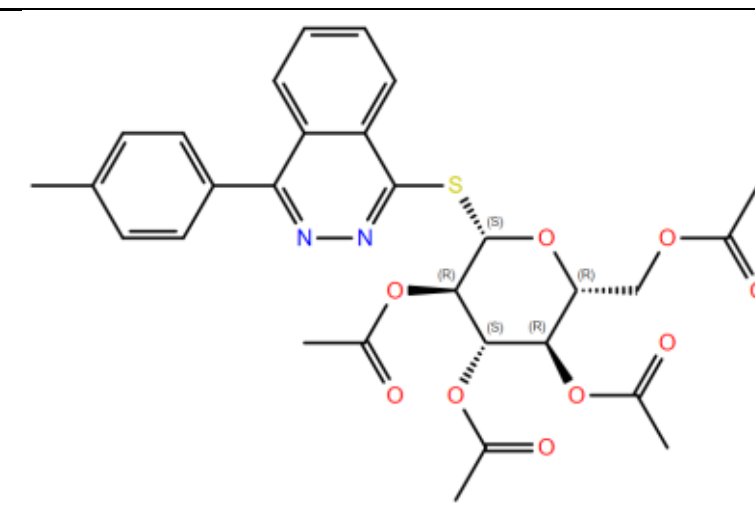 8.635
Compound42	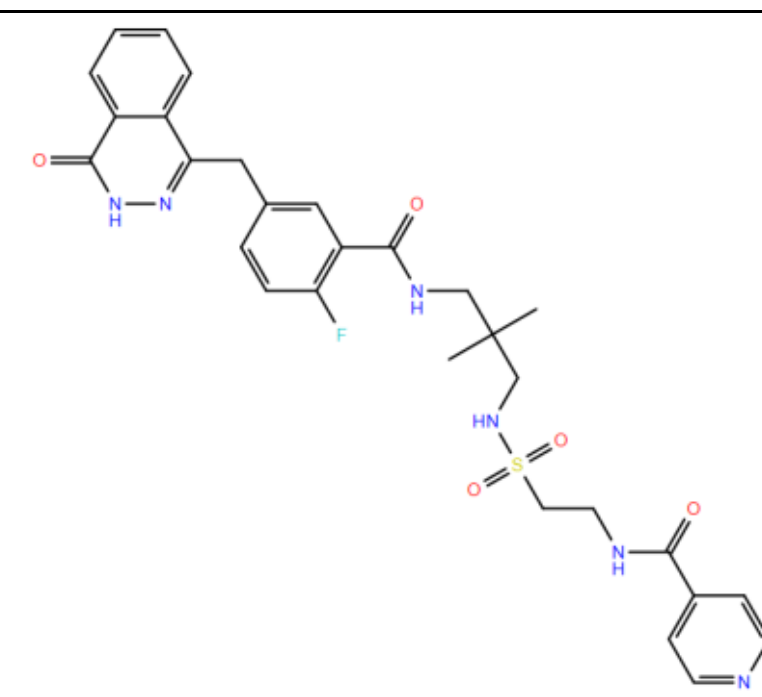 8.440
Compound63	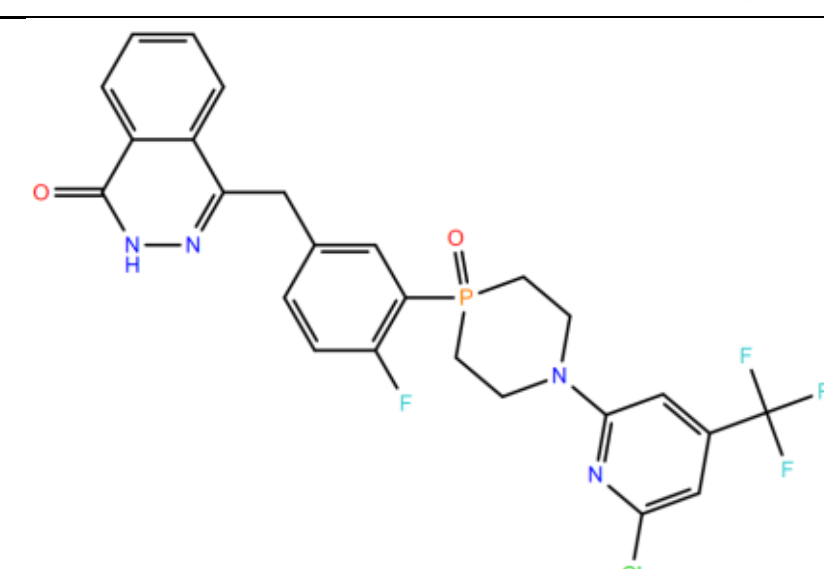 8.336
